# Improving Genotype Imputation in High‐Dimensional Pharmacogenomics Using Multiple Imputation: Evaluation with Machine Learning Approaches

**DOI:** 10.1002/cpt.70171

**Published:** 2025-12-17

**Authors:** Innocent G. Asiimwe, Tao You, Daniel F. Carr, Munir Pirmohamed, Geraint Davies, Andrea L. Jorgensen, the UNITE4TB Consortium

**Affiliations:** ^1^ Department of Health Data Science, Institute of Population Health Sciences University of Liverpool Liverpool UK; ^2^ Department of Pharmacology and Therapeutics, Institute of Systems, Molecular and Integrative Biology University of Liverpool Liverpool UK; ^3^ Department of Clinical Infection, Microbiology & Immunology, Institute of Infection, Veterinary & Ecological Sciences University of Liverpool Liverpool UK

## Abstract

Multiple imputation is well‐established for handling missing data, yet its use in high‐dimensional genetic datasets remains limited. Using pharmacokinetic tuberculosis simulations and SNP data (1000 Genomes Project), we compared machine learning (ML) and traditional approaches (e.g., mean imputation and complete‐case analysis) for imputation and covariate selection. We developed a multiple imputation framework incorporating genotype probabilities, imputation uncertainty (INFO score), and missingness percentages. Dimensionality reduction enabled scalable random forest and penalized regression for covariate selection. In simulations, only multiple imputation achieved adequate coverage (percentage of 95% confidence intervals containing the true value) exceeding a 90% nominal threshold. For example, on the imputation server, coverage improved from 0% with single imputation to up to 94% under 10% missingness. Applied to clinical warfarin datasets (War‐PATH, *n* = 548; IWPC, *n* = 316) and the UK Biobank (*n* = 500, 1000), multiple imputation recovered known pharmacogenomic associations (*CYP2C9* *8/*9/* *11; VKORC1 ‐1639G>A*), reduced false‐positives, and detected signals missed by single imputation (e.g., genome‐wide significant rs4697699, *SLC2A9* locus). Computational costs were modest, adding only ~1.25 minutes for 10 imputations to the 22.7 minutes required by single imputation on the Michigan Imputation Server. For SNP selection, penalized regression performed best in the high‐effect scenario (F1 = 0.897 ± 0.091), while GWAS followed by random forest performed best in the low‐effect scenario (F1 = 0.657 ± 0.110). These findings show that multiple imputation improves reliability and discovery in high‐dimensional pharmacogenomics, with ML offering promising but inconsistent benefits during SNP selection. However, generalizability beyond the studied datasets and computational scalability to larger biobank‐scale analyses remain important limitations that warrant further investigation.

**Study Highlights**

**WHAT IS THE CURRENT KNOWLEDGE ON THE TOPIC?**

Multiple imputation is widely used to address missing data but has rarely been applied to high‐dimensional genetic datasets.

**WHAT QUESTION DID THIS STUDY ADDRESS?**

Can multiple imputation improve accuracy in high‐dimensional genetic analyses, and can machine learning and dimensionality reduction aid covariate selection compared with traditional approaches?

**WHAT DOES THIS STUDY ADD TO OUR KNOWLEDGE?**

Using simulated data, we showed that multiple imputation can be applied to high‐dimensional genetic data with accuracy, achieving adequate coverage (percentage of 95% confidence intervals containing the true value) unlike single‐imputation methods. In clinical warfarin datasets (War‐PATH, IWPC) and the UK Biobank, multiple imputation recovered known pharmacogenomic associations, reduced false‐positives, and detected signals missed by single imputation, with minimal additional computational cost. For covariate selection, machine learning showed potential but was less consistent than genome‐wide association studies (GWASs) and requires further optimization before routine application.

**HOW MIGHT THIS CHANGE CLINICAL PHARMACOLOGY OR TRANSLATIONAL SCIENCE?**

Applying multiple imputation in high‐dimensional genetic settings can improve the reliability of pharmacogenomic discoveries. These findings will inform a planned GWAS sub‐study of the PARADIGM4TB phase IIB/IIC trial evaluating the pharmacokinetics, safety, and efficacy of multiple anti‐tuberculosis drugs.

Multiple imputation is a well‐established method for covariate imputation, but its applicability to high‐dimensional datasets remains limited.^1^ Similarly, traditional pharmacometrics approaches, such as stepwise covariate selection (SCM) are inefficient for analyzing high‐dimensional data.^2^, ^3^ Meanwhile, high‐dimensional data, including clinicogenomic information, is increasingly being used to answer key clinical pharmacology questions in drug development, particularly in optimizing treatment and dosing regimens.^4^ For example, the UNITE4TB consortium (https://www.unite4tb.org/), launched in 2021 with 30 partners from 13 countries, aims to accelerate tuberculosis (TB) treatment development.^5^ Its PARADIGM4TB phase IIB/IIC platform trial is evaluating the pharmacokinetics, safety, and efficacy of multiple drugs, including pyrazinamide, moxifloxacin, bedaquiline, delamanid, linezolid, pretomanid, BTZ‐043, and GSK‐656 (“Ganfeborole”). A sub‐study of this trial will explore pharmacogenetic influences on drug response, incorporating genome‐wide association studies (GWAS) to analyze millions of single‐nucleotide polymorphisms (SNPs).

Genetic data imputation is computationally demanding and typically requires high‐performance computing clusters and at least one genome reference panel.^6^ Servers like the Michigan Imputation Server 2 (MIS2, https://imputationserver.sph.umich.edu/#!) enable genotype imputation for researchers without such infrastructure.^7^ MIS2 generates Variant Call Format (VCF) files^8^ that include Genotype Probability (GP) data (posterior probabilities for each possible genotype at each SNP). These probabilities can be used in multiple imputation methods that account for uncertainty. However, most researchers use the genotype (GT) format, which assigns a single best‐guess genotype per SNP, effectively producing a singly imputed dataset that ignores uncertainty. This simplification can introduce bias and reduce the accuracy of downstream analyses, as single imputation has been shown to be inferior to multiple imputation.^9^, ^10^

Previous studies have evaluated imputation methods for missing covariate data using simulations, comparing single and multiple imputation approaches, simple statistical techniques (e.g., complete‐case analysis/list‐wise deletion, mean imputation), and machine learning (ML) methods (e.g., random forest regression) across the three missing data mechanisms: missing completely at random (MCAR), missing at random (MAR), and missing not at random (MNAR).^11^, ^12^, ^13^, ^14^ However, these studies typically focus on a single missing covariate, with the developers of the Multivariate Imputation by Chained Equations (MICE) R package recommending limiting imputation to subsets of data containing no more than 15–25 variables.^9^, ^10^ As a result, genetic data has yet to fully benefit from the strengths of multiple imputation.

After imputing missing covariate data, appropriate and efficient covariate selection is crucial; however, traditional pharmacometrics approaches such as SCM can be inaccurate, and depending on model complexity, may struggle to handle even a few dozen covariates.^2^, ^3^ Studies by Sibuede *et al*.^2^ and Asiimwe *et al*.^3^ have demonstrated the utility of ML in covariate selection using simulated datasets. These studies recommend ML as a prescreening tool to reduce covariates to a manageable subset before applying traditional pharmacometrics techniques such as SCM, which provide greater interpretability, an essential component during model development. In a previous study, up to 33 covariates were evaluated,^3^ which is far fewer than the millions of SNPs typically available in GWASs.

This study, therefore, compared ML and traditional GWAS approaches in high‐dimensional datasets, evaluating their performance in data imputation, covariate selection, and computational efficiency. Specifically, we (a) implemented a novel multiple imputation strategy using genotype probabilities, imputation uncertainty (INFO score) and missingness percentages from imputation server VCF outputs; (b) applied MICE’s built‐in methods, including the random indicator approach for MNAR data and the quickpred() function to enable imputation of genetic data; (c) validated the imputation approach in clinical datasets; and (d) applied ML and dimensionality reduction techniques for covariate selection in an exploratory manner to assess their potential utility in high‐dimensional settings.

## METHODS

**Figure 1:**
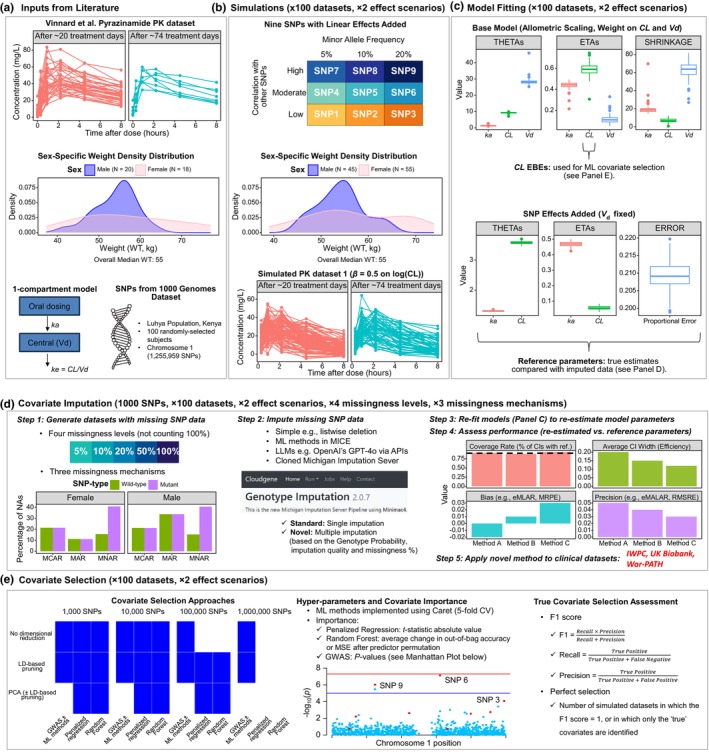
Analysis workflow. (**a**) Inputs. A published Pyrazinamide PK model with SNP data from the 1000 Genomes Luhya population informed the analysis. (**b**) Simulations. Using simulated sex‐specific weights and SNP effects for nine variants, we created 200 PK datasets: 100 with a high effect (*β* = 0.5 on log(CL), ~65% increase in CL) and 100 with a low effect (*β* = 0.15 on log(CL), ~16% increase). (**c**) Model fitting. Each simulated dataset was fitted twice: (i) a base model with allometric scaling on CL and *V*_d_ (to guide ML‐based covariate selection), and (ii) a model including SNP effects (to generate reference parameters for imputation evaluation). (**d**) Covariate imputation. For each simulated dataset, 1000 SNPs (including the nine true ones) were evaluated under four missingness levels and three mechanisms (MCAR, MAR, MNAR). Imputation methods included listwise deletion, ML‐based approaches (MICE R package), server‐based imputation, and a large‐language model. Re‐fitted models were compared with reference values using metrics like the coverage rate, before the novel server‐based multiple imputation method was applied to clinical datasets. (**e**) Covariate selection. Two ML methods and a GWAS approach were applied to SNP sets (1000–1,000,000), with/without LD pruning and PCA. Performance was assessed by F1‐score and perfectly selected datasets. API, application programming interface; CI, confidence interval; CL, clearance (L/hour); CV, cross‐validation; EBE, empirical Bayes estimates; eM(A)LAR, exponent of mean (absolute) log accuracy ratio; ETA, individual random effect (inter‐individual deviation from the population mean); GPT, generative pretrained transformer; GWAS, genome‐wide association study; IWPC, International Warfarin Pharmacogenetics Consortium; *k*_a_, elimination rate constant (hour^−1^); LD, linkage disequilibrium; LLM, large‐language model; MAR, missing at random; MCAR, missing completely at random; MICE, multivariate imputation by chained equations; ML, machine learning; MNAR, missing not at random; MRPE, mean relative prediction error; MSE, mean squared error; *N*, sample size; PCA, principal component analysis; PK, pharmacokinetic; RMSRE, root mean square relative error; SNP, single‐nucleotide polymorphism; THETA, population mean parameter estimate; *V*_d_, volume of distribution (L); War‐PATH, WARfarin anticoagulation in PATients in Sub‐SaHaran Africa; WT, weight; *β*, effect size.

The modeling and simulation analysis (**Figure** **1**), is built upon previous frameworks.^2^, ^3^, ^11^, ^12^, ^13^ All code is available at https://github.com/iasiimwe/unite4tb_ml.

### Literature inputs

For simulation, we used a published^15^ pyrazinamide pharmacokinetic (PK) dataset from a prospective cohort of 40 HIV/TB patients in Gaborone, Botswana. A one‐compartment model with first‐order elimination best described this dataset: elimination rate constant (*k*_a_) = 1.31 hour^−1^, apparent oral clearance (CL) = 3.52 L/hour, and apparent central volume of distribution (*V*_d_) = 28.57 L, using a combined additive (2.41 μg/mL) and proportional error (0.22) model. Allometric scaling with sex‐specific weights (simulated from the original data, median 55 kg) was applied to CL (exponent 0.75) and *V*_d_ (exponent 1).

Sex proportions and SNP data were obtained from the 1000 Genomes Project Phase III project (Luhya population, Webuye, Kenya).^7^ From 117 samples, 100 were randomly selected (55% females). After applying genotype quality control filters (genotyping rate ≥ 95%, minor allele frequency (MAF) ≥ 1%, Hardy–Weinberg Equilibrium *P* < 1 × 10^−6^), 1,255,959 Chromosome 1 SNPs were retained, with up to 1,000,000 used for analysis (**Figure** **S1**).

### Simulated PK datasets

Using parameter estimates and sampling times from Vinnard *et al*.’s structural model,^15^ we simulated PK datasets incorporating sex‐specific weights (for allometric scaling) and SNP effects on CL. Because effect size influences selection accuracy,^2^, ^3^ effect size for log(CL) was set to either small (*β* = 0.15, $\left(\exp\left(\beta \right)-1\right)\times 100\%\approx 16\%$ increase in CL) or large (*β* = 0.5, ~65% CL increase). We generated 100 datasets for each of these two effect size scenarios.

Nine “true” SNP covariates (SNP 1: SNP 9) were assumed to influence CL, modeled using a linear framework with dominant inheritance: heterozygotes and mutant homozygotes exerted equal effects, while wild‐type homozygotes had none. SNPs were selected based on MAF (5%, 10%, 20%) and linkage disequilibrium (LD), stratified into low (no SNPs with *R* ^2^ > 0.1), medium (no SNPs with *R* ^2^ > 0.5), or high (*R* ^2^ > 0.5) correlation structures (**Figure** **S1**).

Simulations were run in Monolix® (versions 2023R1/2024R1) via R using the lixoftConnectors package.^16^

### A priori “true” models

For each scenario and simulated PK dataset, the *a priori* “true” model was re‐estimated twice in Monolix. First, a base model with allometric scaling on CL and *V*_d_ was fitted to generate empirical Bayes estimates (EBEs) for CL, which were used for ML‐based covariate selection. As shown in **Figure** **1** **c**, minimal shrinkage supported their suitability. Second, a model with *V*_d_ fixed and SNP effects included was fitted to obtain reference parameters for evaluating the imputation methods.

### Missing SNP data and covariate imputation

For each scenario and simulated PK dataset, missing data were introduced across 1000 SNPs under four missingness levels (5%, 10%, 20%, and 50%) and three mechanisms: MCAR, MAR, and MNAR. All 991 “false” SNPs followed MCAR, while the nine “true” SNPs were assigned mechanism‐specific missingness probabilities, adapted from Johansson and Karlsson.^12^ Under MCAR, all individuals had equal probability of missingness; under MAR, males were three times more likely than females (83% vs. 27%) to have missing SNPs; under MNAR, mutant‐allele carriers were three times more likely than wild‐types.

We tested a range of imputation strategies, from simple methods (e.g., complete‐case analysis/list‐wise deletion, mode imputation) to advanced techniques using the MICE R package.^9^, ^10^ Tested MICE multiple imputation methods included: Predictive Mean Matching (“pmm”), Weighted PMM (“midastouch”), Random Sampling (“sample”), Classification and Regression Trees (“cart”), Random Forest (“rf”), Unconditional Mean (“mean”), Bayesian Linear Regression (“norm”), and Random Indicator for Nonignorable Data (“ri”). To manage dimensionality, each imputation model included sex, the nine true SNPs, and 20 randomly selected SNPs; the quickpred() function was then used to remove noninformative SNPs, always retaining sex and the nine true SNPs. The number of imputed datasets was equal to the missingness percentage, and final estimates were pooled using Rubin’s rules, as the parameters were approximately normally distributed (confirmed in preliminary runs; **Figure** **S2**).^14^, ^17^, ^18^

We also applied the standard SNP‐imputation workflow using a locally hosted instance of the Michigan Imputation Server 2 (MIS2),^6^ with the 1000 Genomes Phase III African reference panel^7^ [of note, as part of the server‐based quality control process, SNPs with high missingness are typically excluded prior to imputation, introducing in a missingness level of 100% for those SNPs]. Alongside, single imputation using best‐guess genotypes (GT format, where the most probable genotype is selected directly from the imputed probabilities), we developed a multiple imputation approach that builds directly on MIS2 outputs; specifically, genotype probabilities (GP format), imputation uncertainty (INFO score, Rsq), and preimputation missingness (*M*). For each SNP, genotype probabilities (e.g., “0.9, 0.1, 0.0”) were perturbed using a Gaussian distribution with mean zero and standard deviation *U* = (1 − Rsq) × *M*. When either Rsq = 1 or *M* = 0, *U* = 0 and the original probabilities are used without modification, producing consistent genotypes across imputations. When *U* > 0, variability is introduced across imputations, with greater differences arising from lower Rsq or higher *M*. Perturbed probabilities were normalized using min–max scaling and rescaled to sum to one before sampling a genotype (0 = wild‐type homozygote, 1 = heterozygote, 2 = mutant homozygote) via sample (c(0, 1, 2), size = 1, prob = …).

Given rising interest in large‐language models (LLMs) for imputation,^19^ in exploratory work, we also tested OpenAI’s GPT‐4o via an application programming interface on selected datasets, using a structured prompt (see **Text** **S1**).

Each imputed dataset was used to re‐estimate the model, including SNP effects, with a focus on recalculating key pharmacokinetic parameters (e.g., CL). These re‐estimated parameters were then compared with the reference values. Evaluation focused on coverage rate, defined as the percentage of 95% confidence intervals (CIs) containing the true/reference value, with a target of ≥ 90%.^9^ We also assessed average CI width (as a measure of statistical efficiency) and other bias and precision metrics, more applicable to prediction contexts, but commonly used for imputation evaluation (see **Text** **S2**).

To evaluate our server‐based multiple imputation strategy, we applied it to three previously analyzed clinical datasets using an additive genetic model consistent with the original studies. In both studies, the original analyses had relied on single imputation (i.e., best‐guess genotypes). We compared results from our multiple imputation approach to those obtained using this traditional approach. The first was the WARfarin anticoagulation in PATients in Sub‐SaHaran Africa (War‐PATH) cohort (clinical dataset 1), which included 548 warfarin‐treated patients from Uganda and South Africa,^20^, ^21^ focusing on two key pharmacogenes: cytochrome P450, family 2, subfamily C, polypeptide 9 (*CYP2C9*, involved in warfarin’s pharmacokinetics) and vitamin K epoxide reductase complex subunit 1 (*VKORC1*, warfarin’s pharmacological target). The second dataset (clinical dataset 2) was the International Warfarin Pharmacogenetics Consortium (IWPC),^21^, ^22^ comprising 316 warfarin‐treated African Americans. Last, the third dataset (clinical dataset 3) comprised random subsamples (*n* = 500, 1000) from 95,493 unrelated hypertensive, White British, UK Biobank participants, with analysis focused on solute carrier family 2 member 9 (*SLC2A9*), a gene linked to serum urate levels.^23^ Full cohort details are provided in the original publications.^20^, ^21^, ^23^

### Covariate selection for the a priori “true” models

Details on the exploratory covariate selection for the *a priori* “true” models are provided in **Text** **S3** and **Figures** **S3** **and** **S4**. These include two ML algorithms (random forest and penalized regression, implemented in the Caret R package v7.0.1),^24^ traditional GWAS using Monolix‐derived individual random effects (ETAs) for CL as the outcome, dimensionality reduction techniques (PLINK’s^25^ linkage disequilibrium (LD)‐based tag‐SNP selection and principal component analysis), covariate ranking, performance evaluation (F1 score), and runtime assessments.

## RESULTS

### Covariate imputation

#### Only multiple imputation achieves adequate coverage (≥ 90%)

**Figure 2:**
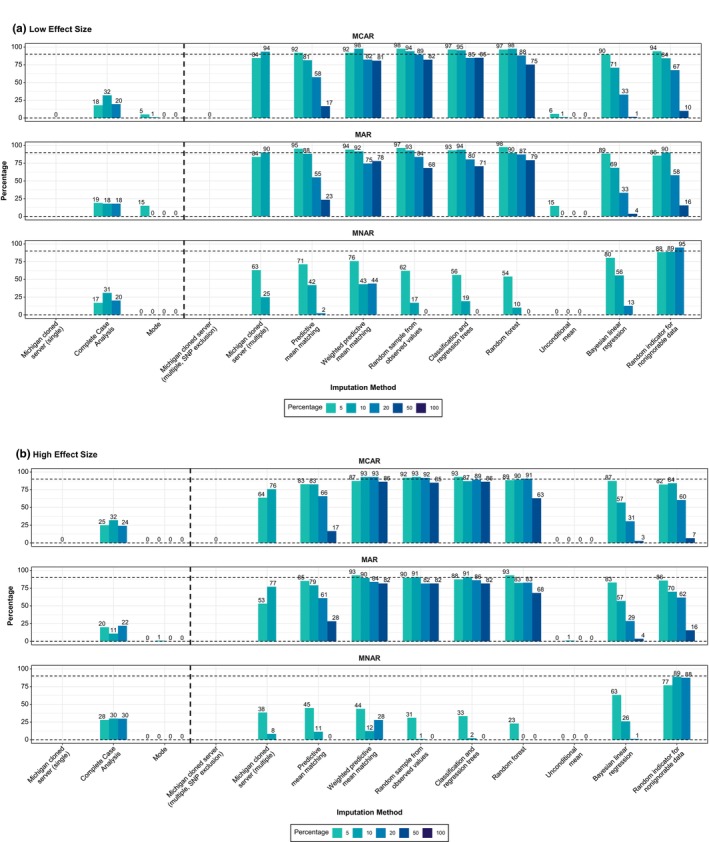
Coverage rates of clearance estimates across imputation methods, missing data mechanisms, and effect sizes. Coverage was defined as the percentage of simulated datasets in which the 95% CI for clearance included the true (reference) value. Panels show results for (**a**) low‐ and (**b**) high‐effect size scenarios, each stratified by missingness mechanism: missing completely at random, missing at random, and missing not at random. Bars represent different imputation methods, with colors indicating the percentage of missing SNP data (5–100%). The horizontal dashed line denotes the nominal 90% coverage threshold, and the vertical dashed line separates single from multiple imputation methods. Numbers above the bars indicate the coverage rate achieved; the absence of a number indicates that the method was not evaluated under that condition. For example, the Michigan server (single imputation) excluded SNPs with high missingness (i.e., 100% missingness for those SNPs) and was therefore evaluated only once. It is displayed only under MCAR, as SNP exclusion renders the missingness mechanism inapplicable. In contrast, the multiple imputation strategy using the Michigan server was assessed at two missingness levels (5% and 10%). CI, confidence interval; MAR, missing at random; MCAR, missing completely at random; MICE, multivariate imputation by chained equations; MNAR, missing not at random; SNP, single‐nucleotide polymorphism.

Across the low‐ and high‐effect scenarios (completed runs in **Figure** **S5**), and for CL coverage rates, only multiple imputation methods consistently achieved the nominal 90% coverage threshold (**Figure** **2**). Among MICE‐based approaches, weighted predictive mean matching, random sampling from observed values, classification and regression trees, and random forest imputation maintained high coverage under MCAR and MAR, often nearing or exceeding the 90% target even at high levels of missingness. While overall performance declined under MNAR, the random indicator for nonignorable data method remained robust, reaching or exceeding 90% in some settings. In contrast, methods such as unconditional mean imputation performed poorly across all mechanisms, with coverage near 0% despite being implemented under a multiple imputation framework.

By comparison, single‐imputation methods, including mode imputation, complete‐case analysis (CCA)/list‐wise deletion, and server‐based single best‐guess genotypes, consistently underperformed. CCA achieved coverage rates of ~25%, while the others had near‐zero coverage across all scenarios. Applying multiple imputation to genotype probabilities and imputation quality metrics (e.g., INFO score values) from the Michigan Imputation Server substantially improved performance; for example, increasing coverage from 0% to 94% under 10% MCAR in the low‐effect setting. However, when SNPs were excluded before imputation (e.g., due to high missingness), no improvement was observed, and coverage remained at 0%. In addition to CL, we evaluated coverage for the elimination rate constant (*k*_a_), as shown in **Figure** **S6**.

#### Narrow confidence intervals (CIs) drive poor coverage rates

**Figure** **S7** shows average CI widths for CL estimates across methods and scenarios. Widths were generally small (< 0.7 in the low‐effect scenario; < 1.7 in the high‐effect scenario), with CCA, mode, and unconditional mean imputation producing especially narrow intervals, which contributed to their poor coverage in **Figure** **2**.

#### Poor bias and precision with SNP exclusion, mode, and unconditional mean imputation

Across all bias metrics (**Text** **S2**), the worst‐performing methods were the server‐based imputation strategy that excluded SNPs prior to imputation, mode imputation, and unconditional mean imputation (**Figures** **S8** **–** **S13**).

#### OpenAI’s GPT‐4o shows bias and precision comparable to mode imputation

In exploratory work, GPT‐4o (“ChatGPT”) produced bias and precision results similar to mode imputation across all metrics (**Figure** **S14**). Log file outputs (panel C) confirmed this, indicating GPT‐4o applied mode imputation when handling missing genotypes.

#### The standard method (imputation server) is the most time‐intensive

**Figure** **S15** shows that server‐based imputation was the slowest method (mean runtime 22.7 minutes), while simple methods like CCA and mode imputation finished in under 10 seconds. MICE‐based methods ranged from 40 seconds to 20 minutes, depending on missingness and number of imputations. Panel C details the Michigan Imputation Server pipeline, where most steps were common to single and multiple imputation; the only added burden was Monolix model fitting (~1.25 minutes for 10 datasets).

#### Server‐based multiple imputation accurately detects known pharmacogenomic associations while reducing false‐positives

**Figure 3:**
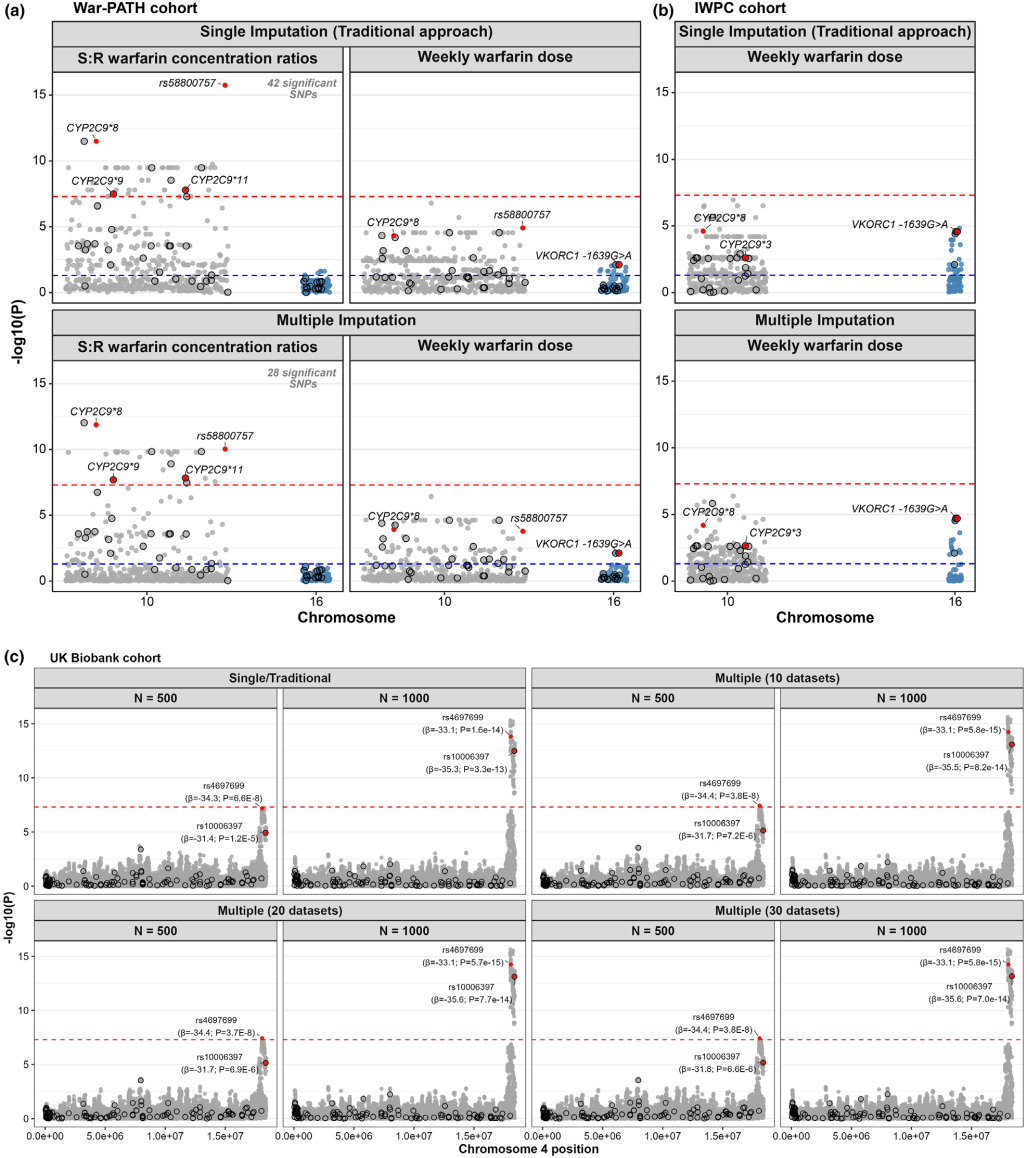
Evaluation of the server‐based multiple imputation strategy on clinical and biobank datasets. (**a**) Manhattan plots for two pharmacogenomic outcomes in the War‐PATH cohort: S:R warfarin concentration ratios (left) and weekly warfarin dose (right), using traditional single imputation (top) and the proposed multiple imputation strategy (bottom). The cohort comprised 548 warfarin‐treated patients from Uganda and South Africa. (**b**) Manhattan plots for weekly warfarin dose in 316 warfarin‐treated African American participants from the IWPC cohort. For both cohorts, analyses focused on *CYP2C9* (chromosome 10) and *VKORC1* (chromosome 16), key pharmacogenes involved in warfarin pharmacokinetics and pharmacodynamics, respectively. Selected variants are annotated; SNPs previously associated with warfarin dosing are highlighted in red, and directly genotyped variants are circled in black. The genome‐wide significance threshold (*P* < 5 × 10^−8^) is indicated by red dashed lines, while an exploratory significance threshold for known associations (*P* < 0.05) is shown as blue dashed lines. (**c**) Manhattan plots for serum urate in random subsets (*N* = 500 and 1000) of unrelated White British hypertensive UK Biobank participants. Association analyses focused on *SLC2A9*, a gene strongly linked to urate regulation. Two SNPs with MAF ~20% are highlighted: one imputed (rs4697699) and one genotyped (rs10006397). *CYP2C9*, cytochrome P450 family 2 subfamily C polypeptide 9; IWPC, International Warfarin Pharmacogenetics Consortium; MAF, minor allele frequency; *N*, sample size; *SLC2A9*, solute carrier family 2 member 9; *VKORC1*, vitamin K epoxide reductase complex subunit 1; War‐PATH, WARfarin anticoagulation in PATients in Sub‐SaHaran Africa; *β*, effect size.

Applying our server‐based multiple imputation strategy to the War‐PATH and IWPC cohorts recovered known pharmacogenomic associations, particularly in *CYP2C9* (*8, *9, and *11) for the S:R warfarin concentration ratio (**Figure** **3**). Importantly, CYP2C9*8, an imputed variant, remained significant, demonstrating the method’s ability to capture true associations from imputed data. In War‐PATH, the top SNP under single imputation was rs58800757, an upstream variant of *LOC107984256*, which showed low LD with CYP2C9*8 (*R* ^2^ = 0.12), *9 (*R* ^2^ = 0.03), and *11 (*R* ^2^ = 0.03), suggesting a false positive. After multiple imputation, rs58800757 became less significant than CYP2C9*8, and the number of top SNPs associated with the S:R ratio decreased from 42 to 28, indicating reduced spurious findings.

For warfarin dose, no SNPs reached genome‐wide significance, likely due to smaller sample sizes (War‐PATH: *n* = 548; IWPC: *n* = 316). However, several variants, including *VKORC1‐1639G>A*, surpassed the exploratory threshold (*P* < 0.05) and showed cleaner signals after multiple imputation in both cohorts, with known associations more clearly distinguished from nearby SNPs likely to be false‐positives.

In addition to inflating statistical significance and increasing false‐positives, single imputation can also deflate significance, causing missed signals (**Figure** **S16**). For example, in **Figure** **3** **c**, multiple imputation with 10 datasets and a sample size of 500 detected genome‐wide significance for the top SNP rs4697699 in the *SLC2A9* locus (*P* = 3.8 × 10^−8^), whereas single imputation failed (*P* = 6.6 × 10^−8^; threshold 5 × 10^−8^). Increasing imputations from 10 to 20 or 30 had marginal gains, indicating little benefit beyond 10 imputations in this particular analysis.

### Exploratory covariate selection

#### Low‐effect signals harder to detect

**Figure 4:**
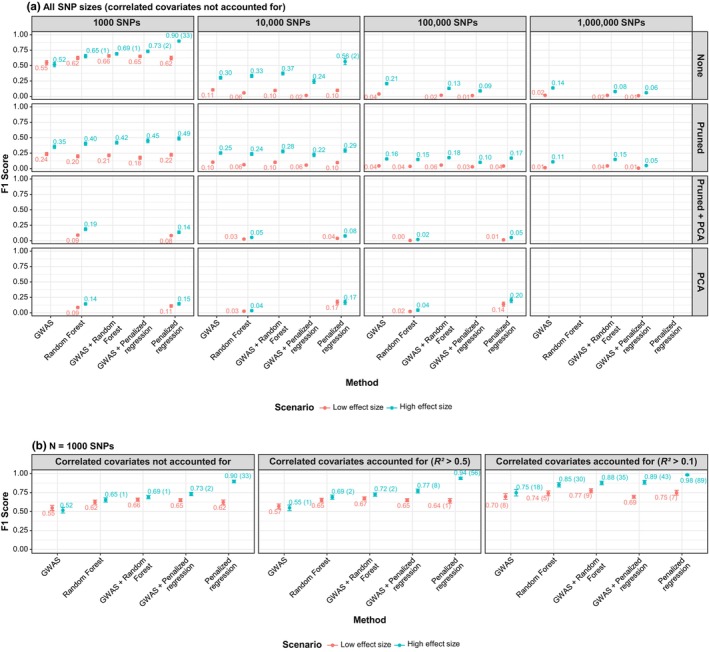
F1 scores for each method, stratified by effect size, SNP count, and dimensionality reduction approach. (**a**) All SNP sizes without adjustment for correlation. (**b**) Subset of 1000 SNPs with no dimensionality reduction, further divided into: (i) no correlation adjustment, (ii) adjustment for correlated covariates with *R* ^2^ > 0.5, and (iii) adjustment with *R* ^2^ > 0.1. For correlated scenarios, a SNP was counted as selected if it or a highly correlated SNP (above the given *R* ^2^ threshold) was selected. Methods are ordered by F1 score (left to right). F1 values are annotated, with numbers in parentheses indicating datasets with perfect selection (F1 = 1). Error bars represent 95% confidence intervals. Population pharmacokinetic individual random effects (ETAs) served as the outcome variable. GWAS, genome‐wide association study; MAF, minor allele frequency; *N*, sample size; PCA, principal component analysis; Pruned, linkage disequilibrium‐based pruning; SNP, single‐nucleotide polymorphism.

**Figure 5:**
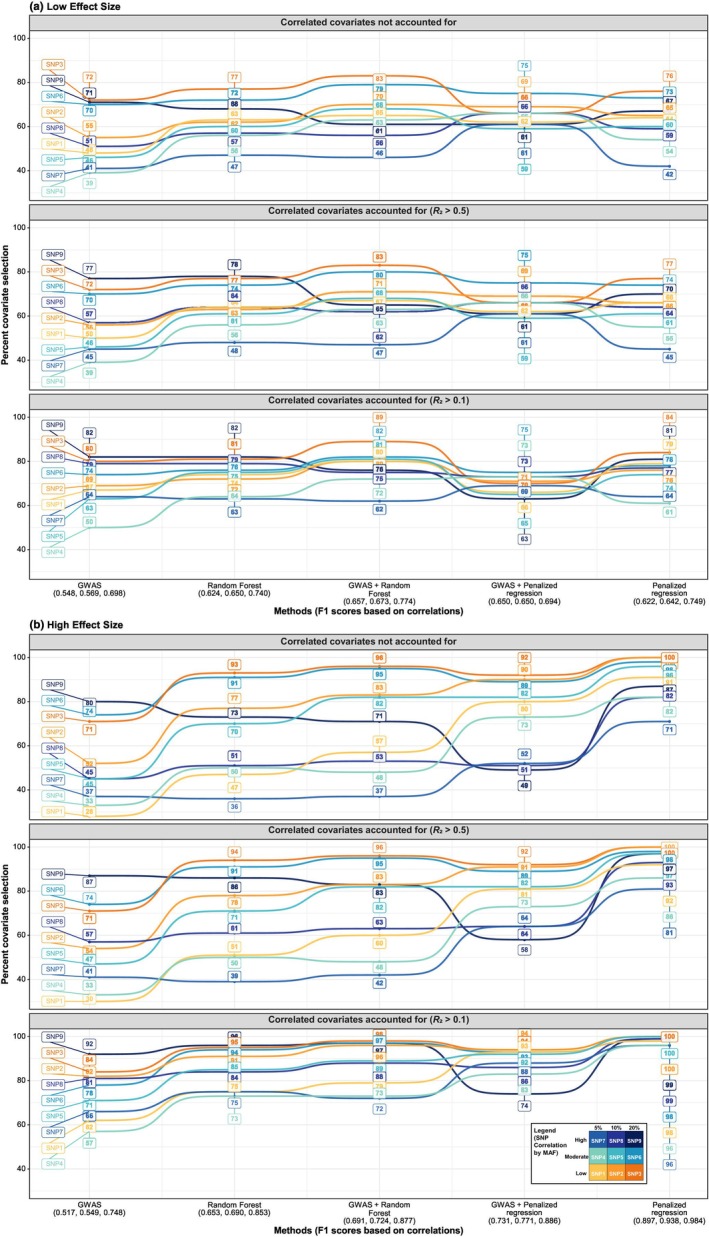
Proportion of true covariates (SNPs) selected by each method, stratified by effect size scenario (1000 SNPs). (**a**) Low‐effect size. (**b**) High‐effect size. Each panel shows covariate selection performance across three conditions, (i) no adjustment for correlation, (ii) adjustment for correlated covariates with *R* ^2^ > 0.5, and (iii) adjustment with *R* ^2^ > 0.1. When adjusting for correlation, a covariate was considered “selected” if it or any of its highly correlated counterparts (above the specified *R* ^2^ threshold) was selected. Methods are ranked left to right based on their F1‐score (shown below the *x*‐axis for the three conditions). Population pharmacokinetic individual random effects (ETAs) served as the outcome variable. GWAS, genome‐wide association study; MAF, minor allele frequency; SNP, single‐nucleotide polymorphism.

As shown in Table 1 and **Figure** **4** **a** (1000 SNPs, no dimensionality reduction), penalized regression performed best in the high‐effect scenario (F1 = 0.897 ± 0.091) but dropped in the low‐effect scenario (F1 = 0.622 ± 0.157). GWAS + random forest performed best for low‐effect covariates (F1 = 0.657 ± 0.110). **Figure** **5** further illustrates this, with penalized regression identifying SNP 3 in all datasets under the high‐effect scenario but only 76% of datasets under the low‐effect scenario.

**Table 1:** F1 scores for each method, stratified by effect size, SNP count, and dimensionality reduction approach

SNP count	Method	Low‐effect size				High‐effect size			
		None	Pruned	Pruned + PCA	PCA	None	Pruned	Pruned + PCA	PCA
1000	GWAS	0.548 (0.167)	**0.236 (0.131)**	NA	NA	0.517 (0.185)	0.353 (0.144)	NA	NA
	Penalized regression	0.622 (0.157)	0.224 (0.126)	0.083 (0.084)	**0.111 (0.111)**	**0.897 (0.091)**	**0.488 (0.138)**	0.137 (0.116)	**0.146 (0.117)**
	Random Forest	0.624 (0.139)	0.199 (0.130)	**0.090 (0.087)**	0.087 (0.089)	0.653 (0.144)	0.403 (0.138)	**0.187 (0.122)**	0.144 (0.101)
	GWAS + Penalized regression	0.650 (0.104)	0.176 (0.133)	NA	NA	0.731 (0.113)	0.450 (0.157)	NA	NA
	GWAS + Random Forest	**0.657 (0.110)**	0.214 (0.118)	NA	NA	0.691 (0.112)	0.421 (0.128)	NA	NA
10,000	GWAS	**0.107 (0.091)**	**0.104 (0.086)**	NA	NA	0.303 (0.121)	0.253 (0.114)	NA	NA
	Penalized regression	0.099 (0.102)	0.097 (0.087)	**0.038 (0.069)**	**0.173 (0.173)**	**0.564 (0.241)**	**0.293 (0.146)**	**0.077 (0.094)**	**0.169 (0.175)**
	Random Forest	0.058 (0.087)	0.063 (0.079)	0.026 (0.047)	0.026 (0.072)	0.333 (0.136)	0.236 (0.124)	0.053 (0.071)	0.036 (0.083)
	GWAS + Penalized regression	0.016 (0.042)	0.056 (0.078)	NA	NA	0.244 (0.173)	0.223 (0.153)	NA	NA
	GWAS + Random Forest	0.097 (0.092)	0.103 (0.091)	NA	NA	0.373 (0.127)	0.279 (0.126)	NA	NA
100,000	GWAS	**0.040 (0.066)**	**0.044 (0.059)**	NA	NA	**0.208 (0.103)**	0.157 (0.088)	NA	NA
	Penalized regression	NA	0.041 (0.056)	**0.014 (0.038)**	**0.140 (0.176)**	NA	0.170 (0.091)	**0.051 (0.075)**	**0.202 (0.188)**
	Random Forest	NA	0.036 (0.057)	0.004 (0.022)	0.022 (0.079)	NA	0.147 (0.097)	0.019 (0.045)	0.044 (0.109)
	GWAS + Penalized regression	0.014 (0.038)	0.029 (0.054)	NA	NA	0.090 (0.104)	0.102 (0.087)	NA	NA
	GWAS + Random Forest	0.019 (0.045)	0.056 (0.066)	NA	NA	0.131 (0.100)	**0.177 (0.087)**	NA	NA
1,000,000	GWAS	**0.018 (0.047)**	0.014 (0.038)	NA	NA	**0.138 (0.091)**	0.109 (0.086)	NA	NA
	GWAS + Penalized regression	0.012 (0.035)	0.007 (0.027)	NA	NA	0.061 (0.088)	0.049 (0.075)	NA	NA
	GWAS + Random Forest	0.017 (0.043)	**0.043 (0.057)**	NA	NA	0.079 (0.092)	**0.148 (0.081)**	NA	NA

F1 scores are reported as means with standard deviations in parentheses. For each combination of effect size, SNP count, and dimensionality reduction approach, the best‐performing method is bolded. Population pharmacokinetic individual random effects (ETAs) were used as the outcome variable. GWAS, genome‐wide association study; MAF, minor allele frequency; *N*, sample size; NA, not applicable; none, no dimensionality reduction applied; PCA, principal component analysis; Pruned, linkage disequilibrium‐based pruning; SNP, single‐nucleotide polymorphism.

#### Dimensionality reduction did not improve performance

LD‐based pruning, PCA, and combined approaches consistently reduced accuracy (Table 1, **Figure** **4** **a**). For example, in the 1000‐SNP low‐effect scenario, penalized regression F1 dropped from 0.622 ± 0.157 (no reduction) to 0.224 ± 0.126 (pruning), 0.083 ± 0.084 (pruning + PCA), and 0.111 ± 0.111 (PCA). Similar trends were observed in the high‐effect scenario. Dimensionality reduction, however, enabled ML methods to scale to 100,000 SNPs and reduced runtime (e.g., random forest from 12 to < 3 minutes in the 10,000‐SNP subset, **Figure** **S17**).

#### Performance declined with increasing SNPs

Without dimensionality reduction, GWAS F1 scores fell from 0.548 ± 0.167 at 1000 SNPs to 0.018 ± 0.047 at 1 million SNPs (low‐effect scenario). This trend was also seen in the high‐effect scenario and across other methods with/without dimensionality reduction (Table 1, **Figure** **4** **a**).

#### Accounting for correlation improves accuracy

Accounting for correlations among covariates, by considering a SNP as correctly selected if it or a highly correlated SNP was identified, increased penalized regression F1 from 0.622 (no correlation) to 0.642 (*R* ^2^ > 0.5) and 0.749 (*R* ^2^ > 0.1) in the low‐effect scenario, and from 0.897 to 0.938 and 0.984 in the high‐effect scenario (**Figures** **4** **b** **and** **5**). This was similar for other methods.

#### Common, uncorrelated SNPs are easiest to detect

As shown in **Figure** **5**, SNP 3 (MAF = 20%, low correlation) was the most consistently selected, identified by penalized regression in 76% of low‐effect and all high‐effect datasets. SNPs 6 and 9 (MAF = 20%) were also frequently selected. Accounting for correlations increased selection of highly correlated SNPs; for example, for GWAS in the low‐effect scenario, SNP 9 rose from 71% to 77% when *R* ^2^ > 0.5 was applied, whereas SNP 3 remained at 72%.

#### Random forest and penalized regression identify some known signals when applied to clinical/biobank data

**Figure 6:**
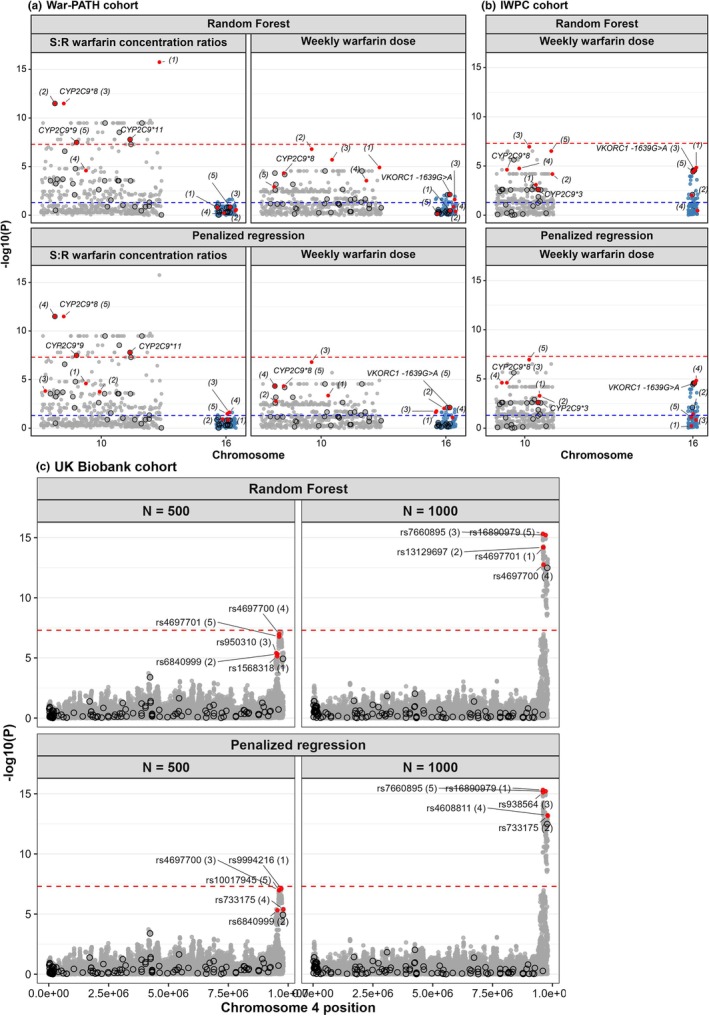
Application of covariate selection methods to clinical and biobank datasets. (**a**) Manhattan plots for two pharmacogenomic outcomes in the War‐PATH cohort: S:R warfarin concentration ratios (left) and weekly warfarin dose (right), analyzed using random forest (top) and penalized regression (bottom) after GWAS‐based preselection of the top 1000 SNPs. The cohort included 548 warfarin‐treated patients from Uganda and South Africa. (**b**) Manhattan plots for weekly warfarin dose in 316 African American participants from the IWPC cohort. Analyses in both a and b focused on two key warfarin‐related pharmacogenes: *CYP2C9* (chromosome 10; pharmacokinetics) and *VKORC1* (chromosome 16; pharmacodynamics). (**c**) Manhattan plots for serum urate levels in random subsets (*N* = 500 and *N* = 1000) of unrelated White British hypertensive participants from the UK Biobank, with analyses targeting the *SLC2A9* locus on chromosome 4, a gene strongly implicated in urate transport. In all panels, the five top‐ranked SNPs (based on random forest or penalized regression importance scores) are highlighted in red and annotated with their ranks in parentheses. SNPs previously associated with the traits are also highlighted in red, and directly genotyped variants are circled in black. The genome‐wide significance threshold (*P* < 5 × 10^−8^) is indicated by red dashed lines, and an exploratory threshold for known associations (*P* < 0.05) is shown in blue (**a**–**b** only). *CYP2C9*, cytochrome P450 family 2 subfamily C member 9; GWAS, genome‐wide association study; IWPC, International Warfarin Pharmacogenetics Consortium; *N*, sample size; *SLC2A9*, solute carrier family 2 member 9; *VKORC1*, vitamin K epoxide reductase complex subunit 1; War‐PATH, WARfarin anticoagulation in PATients in Sub‐SaHaran Africa.

Applying random forest and penalized regression directly to SNP sets > 1000 was computationally intensive, but both became feasible after GWAS preselection of the top 1000 SNPs (as done in **Figure** **6**). In War‐PATH, random forest ranked CYP2C9*8 and *9 among the top five for the S:R warfarin ratio, and penalized regression also ranked CYP2C9*8 highly, along with *VKORC1 ‐1639G>A* for weekly dose. In IWPC, random forest ranked *VKORC1 ‐1639G>A* among the top five, while penalized regression ranked CYP2C9*8 as a top five variant. In the UK Biobank, penalized regression ranked rs16890979 (a missense *SLC2A9* variant) first and random forest ranked it fifth with *n* = 1000.

## DISCUSSION

Genetic data are inherently high‐dimensional, often comprising millions of SNPs, making analysis computationally intensive. Consequently, most, if not all, previous GWASs have relied on imputation servers and single imputation using best‐guess genotypes. However, single imputation is known to be biased and less robust than multiple imputation.^9^, ^10^ In this study, we extended multiple imputation to genetic data by incorporating genotype probabilities, imputation quality metrics (INFO score), and missingness percentages from an imputation server, and evaluated the approach in both simulations and clinical datasets.

In the controlled setting, multiple imputation consistently outperformed single imputation, particularly in coverage, the probability of containing the true value within 95% confidence intervals. Applied to two clinical warfarin datasets and the UK Biobank, it recovered established pharmacogenomic associations, reduced false‐positives, and detected signals missed by single imputation. Importantly, the workflow remains largely compatible with existing pipelines, as data preparation, server‐side imputation, and result extraction are the same as for single imputation. The only added step, analyzing multiply imputed datasets, does not substantially increase processing time.

A common GWAS quality control step is to exclude SNPs with low genotyping rates (< 95–99%) due to reliability concerns.^26^ However, our simulations show that excluding such SNPs discards potentially recoverable information; multiple imputation on datasets with excluded SNPs performed no better than single imputation. This supports the principle that incomplete data, if properly handled, is preferable to omission. Although our real‐world datasets had already excluded low‐quality SNPs, our results suggest that incorporating SNP quality metrics, such as missingness percentage, into imputation is both feasible and beneficial.

Large‐language models (LLMs) such as OpenAI’s GPT‐4o have gained popularity for tasks like data imputation.^19^ During exploratory evaluation, GPT‐4o produced results nearly identical to mode imputation, likely reflecting reliance on common methods in its training data rather than optimal choices. Of note, mode imputation consistently underperformed in our analyses, even within multiple imputation, highlighting the limitations of GPT‐4o’s choice. While LLMs provide user‐friendly interfaces and tools (e.g., OpenAI’s code interpreter, which we used to streamline analysis), their accessibility can be a double‐edged sword if nonexperts over‐trust outputs without recognizing these limitations. As LLMs evolve, they may incorporate more appropriate statistical methods for domain‐specific tasks like genetic multiple imputation, warranting continued evaluation of their utility in covariate imputation and related applications.

In controlled simulations, covariate selection techniques reaffirmed known patterns: low‐effect signals are harder to detect, higher dimensionality reduces accuracy, correlated covariates improve performance when accounted for, and common, uncorrelated SNPs are easiest to detect.^2^, ^3^ Dimensionality reduction lowered performance, likely from loss of informative SNPs, but allowed computationally intensive methods like random forest and penalized regression to scale to 100,000 SNPs. These ML approaches also became feasible when applied to GWAS‐preselected SNPs. In clinical warfarin datasets and the UK Biobank, results were mixed; while some established associations were successfully identified, results varied across datasets and methods, highlighting the need for further optimization of covariate selection in high‐dimensional genomic analyses.

A key strength of this study is the application of a multiple imputation framework to both simulated and real‐world (clinical/biobank) genetic data. To ensure compatibility with existing genotype imputation pipelines that account for the uniqueness of genetic data (being constrained by principles such as Hardy–Weinberg equilibrium (HWE), linkage disequilibrium (LD), and haplotype structure), our multiple imputation approach was designed to extend rather than replace the standard workflow. In real‐world analyses, we focused on established associations to validate the approach, which limited our ability to assess the full computational demands of genome‐wide multiple imputation. Still, we showed that its workflow mirrors single imputation in all major steps, differing only in downstream analysis of multiply imputed datasets, a step now readily manageable with parallel computing, removing a common justification for single imputation. As a reminder, researchers should ensure high‐quality input data by following the preparation steps recommended by the Michigan Imputation Server 2 (MIS2), as well as good methodological practices outlined in the STrengthening the Reporting Of Pharmacogenetic Studies (STROPS) guideline.^27^ Although standard workflows such as the MIS2 account for the uniqueness of genetic data, limitations remain, and future work is needed to fully integrate HWE and LD constraints directly within multiple imputation frameworks.

Another limitation is that we did not report results for some MICE methods (e.g., *lasso.norm*, *lasso.select.norm*), which generated persistent collinearity‐related warnings driven by the strong correlations inherent in genetic data. Because these warnings reflected methodological instability rather than problems with the data itself, we opted to report only those approaches that ran robustly and produced reliable imputations. We also evaluated deterministic methods such as unconditional mean imputation, in which the completed datasets produced across MICE iterations are identical. Because these approaches introduce no stochastic variation, they effectively function as single‐imputation methods despite being executed within a multiple imputation framework. We also recognize that our imputation models may have excluded relevant variables, including the outcome.^28^ This decision was driven by pragmatic constraints, as it is infeasible to include all relevant variables when imputing millions of SNPs; however, standard SNP‐imputation workflows (e.g., MIS2) could benefit from incorporating additional nongenetic variables, including outcome data, where feasible. Additionally, our analysis focused on fixed effects, which align with normality assumptions; further methodological work is needed to appropriately account for random‐effect variances and residual error when applying multiple imputation approaches. A further limitation is that UK Biobank genotype probabilities were available only for a subset of variants, meaning our results likely represent a minimum benefit; broader availability would likely yield greater improvements. Finally, simulations were based on tuberculosis data; however, validation in independent warfarin and hypertension cohorts supports robustness and generalizability. Nonetheless, generalizability beyond the studied datasets and computational scalability to larger biobank‐scale analyses remain important limitations that warrant further investigation.

In conclusion, this work, conducted in preparation for the PARADIGM4TB phase IIB/IIC sub‐study, reaffirmed the limitations of single imputation (the standard GWAS approach) and introduced a multiple imputation framework that we will adopt moving forward. We also evaluated covariate selection methods, including random forest and penalized regression, which showed some promise but inconsistent performance; for now, we will continue to rely on traditional GWAS. Future efforts will focus on optimizing imputation and covariate selection to enable faster, more reliable decision making in drug discovery and development.

## FUNDING

This project has received funding from the Innovative Medicines Initiative 2 Joint Undertaking (JU) under grant agreement No 101007873. The JU receives support from the European Union’s Horizon 2020 research and innovation program and EFPIA, Deutsches Zentrum für Infektionsforschung e. V. (DZIF), and Ludwig‐Maximilians‐Universität München (LMU). EFPIA/AP contributes 50% of the funding, whereas the contribution of DZIF and the LMU University Hospital Munich has been granted by the German Federal Ministry of Education and Research.

## CONFLICT OF INTEREST

This project has received funding from the Innovative Medicines Initiative 2 Joint Undertaking (JU) under grant agreement No 101007873. The JU receives support from the European Union’s Horizon 2020 research and innovation program and EFPIA, Deutsches Zentrum für Infektionsforschung e. V. (DZIF), and Ludwig‐Maximilians‐Universität München (LMU). EFPIA/AP contributes 50% of the funding, whereas the contribution of DZIF and the LMU University Hospital Munich has been granted by the German Federal Ministry of Education and Research. M.P. currently receives partnership funding, paid to the University of Liverpool, for the following: MRC Clinical Pharmacology Training Scheme (co‐funded by MRC and Roche, UCB, Eli Lilly and Novartis), and the MRC Medicines Development Fellowship Scheme (co‐funded by MRC and GSK, AZ, Optum and Hammersmith Medicines Research). He has developed an HLA genotyping panel with MC Diagnostics but does not benefit financially from this. He is part of the IMI Consortium ARDAT (www.ardat.org); none of these of funding sources have been used for the current research. D.C. currently receives research funding from ViiV health care. I.G.A. is currently employed by Novartis Pharmaceuticals UK Ltd, with employment commencing after all work described in this paper was completed. All other authors declared no competing interests for this work.

## AUTHOR CONTRIBUTIONS

All authors wrote the manuscript and designed the research. I.G.A. performed the research and analyzed the data. All authors contributed new reagents/analytical tools.

## DISCLAIMER

This communication reflects the authors’ view and neither IMI nor the European Union, EFPIA, or any Associated Partners are responsible for any use that may be made of the information contained therein.

## Supporting information


Data S1

## Data Availability

All data used in the simulation analyses are either included in the code repository or publicly available from the 1000 Genomes Project (ftp://ftp.1000genomes.ebi.ac.uk/vol1/ftp/data_collections/1000_genomes_project/release/20190312_biallelic_SNV_and_INDEL). Access to clinical and biobank datasets requires appropriate permissions: UK Biobank data are available via application (https://www.ukbiobank.ac.uk/), and the War‐PATH and IWPC dataset may be obtained from the authors upon reasonable request and with appropriate approval. All code used in this analysis is publicly available at: https://github.com/iasiimwe/unite4tb_ml.
