# Supplementary material for: Improving Genotype Imputation in High‐Dimensional Pharmacogenomics Using Multiple Imputation: Evaluation with Machine Learning Approaches
Source: Clin Pharmacol Ther. 2025 Dec 17;119(4):964–78. doi: 10.1002/cpt.70171 (PMC12997507; doi:10.1002/cpt.70171)
Supplement: Supplementary file 1 — Data S1 [file CPT-119-964-s001.docx]

**Improving Genotype Imputation in High-Dimensional Pharmacogenomics Using Multiple Imputation: Evaluation with Machine Learning Approaches**

Innocent G. Asiimwe^1^*, Tao You^2^, Daniel F. Carr^2^, Munir Pirmohamed^2^, Geraint Davies^3^, Andrea L. Jorgensen^1^*, on behalf of the UNITE4TB consortium^4^.

^1^Department of Health Data Science, Institute of Population Health Sciences, University of Liverpool, Liverpool, UK.

^2^Department of Pharmacology and Therapeutics, Institute of Systems, Molecular and Integrative Biology, University of Liverpool, Liverpool, UK.

^3^Department of Clinical Infection, Microbiology & Immunology, Institute of Infection, Veterinary & Ecological Sciences, University of Liverpool, Liverpool, UK.

^4^[www.unite4TB.org](http://www.unite4TB.org)

**Correspondence:**

*Correspondence and requests for materials should be addressed to IGA or ALJ (email: [i.asiimwe@liverpool.ac.uk](mailto:i.asiimwe@liverpool.ac.uk), [aljorgen@liverpool.ac.uk](mailto:aljorgen@liverpool.ac.uk)).

**Contents**

**Supplementary Text…………………………………………………………………………………2**

**Supplementary Figures………………………………………………………………..…………….5**

**Supplementary References……………………………………………………………………....…44**

**Supplementary Text**

**Text S1: OpenAI’s GPT-4o structured prompt**

prompt = *"""*

*Task Description:*

*You are an expert data scientist specializing in data analysis and imputation. Your task is to analyze and process a tuberculosis (TB) dataset provided as a CSV file. The dataset contains the following columns:*

*- **Participant ID**: A unique identifier for 100 participants.*

*- **Sex**: Encoded as 1 for male and 2 for female.*

*- **Single Nucleotide Polymorphisms (SNPs)**: Columns labeled SNP1 to SNP1000. Among these, the first nine SNPs (SNP1-SNP9) are the covariates of primary interest.*

*### Missing Data*

*Some SNP data contain missing values, denoted as NaN. The missing data follow three distinct mechanisms:*

*1. **Missing Completely at Random (MCAR)**: Missingness is unrelated to the data.*

*2. **Missing at Random (MAR)**: Missingness is related to other observed data.*

*3. **Missing Not at Random (MNAR)**: Missingness depends on the value of the missing data itself.*

*### Objectives*

*1. **Identify Missingness Mechanisms**:*

*- Determine the missingness mechanism (MCAR, MAR, or MNAR) for each SNP1-SNP9.*

*2. **Impute Missing Values**:*

*- Impute the missing values for SNP1-SNP9 using methods that align with the identified missingness mechanisms.*

*- Ensure all imputed values are either:*

*- `0` (wild-type homozygotes)*

*- `1` (mutant allele).*

*3. **Export Results**:*

*- Display the imputed dataset with the following columns: ID, sex, and SNP1-SNP9.*

*- Include all 100 participants in your output.*

*- SNP1-SNP9 should not have any missing entries.*

*Example:*

*ID sex SNP1 SNP2 SNP3 SNP4 SNP5 SNP6 SNP7 SNP8 SNP9*

*NA19017 2 0 0 0 0 0 1 0 0 0*

*NA19020 1 0 0 1 0 0 0 0 0 0*

*...*

*4. **Report Findings**:*

*- Provide a summary of the identified missingness mechanisms for SNP1-SNP9.*

*- Clearly outline how each imputation method was selected and implemented.*

*- Include a link to download the imputed dataset in the format:*

*`[Download the imputed dataset](sandbox:/mnt/data/imputed_tb_data.csv)`.*

*### Requirements*

*- Imputation methods must strictly correspond to the identified missingness mechanisms.*

*- Generate clean and reproducible code with explanations in comments.*

*- Ensure the output aligns with the given specifications.*

*"""*

**Text S2: Covariate Imputation Bias and Precision Metrics**

Population pharmacokinetic models were re-estimated for each imputed dataset, and the resulting parameter estimates (“Estimated”) were compared against those from the original dataset with no missing data (“True”). Bias metrics assessed systematic over- or underestimation, while precision metrics evaluated the closeness of estimated values to the true values.^1, 2^

In predictive modelling, ratio-based metrics such as the Root Mean Square Relative Error (RMSRE) are generally preferred over absolute measures like Root Mean Square Error (RMSE), as they account for the relative magnitude of the error. For example, a 1-unit error is more clinically significant when the true value is 2 than when it is 10.^3, 4^

The most robust bias and precision measures are derived from the logarithm of the accuracy ratio,^3, 4, 5^ though these are less commonly used in pharmacometrics. Therefore, we also report more conventional metrics.

The bias metrics evaluated were:^1, 2, 3, 4, 5^

- Exponent of the logarithm of the accuracy ratio (eMLAR, measures bias by accounting for multiplicative error), where eMLAR (%) = $(exp(\frac{1}{n}\sum_{i=1}^{n} \log(\frac{{Estimated}_{i}}{{True}_{i}}))-1)\times100$,
- Mean relative prediction error (MRPE, evaluates relative error at the individual prediction level), where MRPE (%) = $\frac{100}{n}\sum_{i=1}^{n} (\frac{{Estimated}_{i}-{True}_{i}}{{True}_{i}})$, and
- Relative mean prediction error (rMPE, provides an overall measure of bias relative to the scale of the true values), where rMPE (%) = $\frac{100}{n \times mean True value}\sum_{i=1}^{n} {(Estimated}_{i}-{True}_{i})$.

On the other hand, the precision metrics were:

- Exponent of the absolute logarithm of the accuracy ratio (eMALAR, quantifies precision by accounting for multiplicative error magnitude), where eMALAR (%) = $(exp(\frac{1}{n}\sum_{i=1}^{n} |log(\frac{{Estimated}_{i}}{{True}_{i}})|)-1)\times100$,
- Root mean square relative error (RMSRE, evaluates the spread of relative errors and is sensitive to outliers), where RMSRE (%) = $(\sqrt{{\frac{1}{n}\sum_{i=1}^{n} (\frac{{Estimated}_{i}-{True}_{i}}{{True}_{i}})}^{2}})\times100$, and,
- Mean absolute percentage error (MAPE, less sensitive to outliers compared to RMSRE), where MAPE (%) $=\frac{1}{n}\sum_{i=1}^{n} |\frac{{Estimated}_{i}-{True}_{i}}{{True}_{i}}|\times100$.

**Text S3: Covariate Selection for the A priori ‘True’ Models**

In preliminary work, we attempted Monolix’s COSSAC-SAMBA (COnditional Sampling use for Stepwise Approach based on Correlation with Stochastic Approximation for Model Building Algorithm) (Figure S3). For up to 500 SNPs, runtime increased cubically with the number of SNPs (line of best fit: *y* = *x³*), meaning that doubling the number of SNPs would result in an eightfold increase in computation time. For instance, analysing 500 SNPs required approximately half a day, with extrapolation suggesting that 1,000 SNPs would take ~4 days, and 1 million SNPs several years. This was deemed computationally infeasible.

Subsequently, we tried two ML algorithms: penalized regression and random forest, implemented via the Caret R package (version 7.0.1).^6^ Hyperparameters (e.g., alpha for penalized regression, number of trees for random forest) were tuned using five-fold cross-validation with default settings. As a third approach, we applied traditional genome-wide association study (GWAS), using Monolix-derived individual random effects (ETAs) for clearance (CL) as the outcome (Figure 1C).

As shown in Figure 1E, only the GWAS approach, which tests SNPs individually, could handle datasets with 100,000 or more SNPs. In contrast, ML methods that analyze all SNPs simultaneously were computationally constrained. To address this, we first applied dimensionality reduction using PLINK’s^7^ linkage-disequilibrium (LD)-based tag-SNP selection (*--indep-pairwise*) with optimized parameters: 1,000 kb window, 5-SNP step, and *r²* threshold of 0.1 (Supplementary Figure 3). While this reduced the 1,000,000-SNP dataset to ~100,000 SNPs, ML methods remained limited. We therefore applied principal component analysis (PCA) and GWAS preselection (top 100–1,000 SNPs) for further reduction. As GWAS requires individual SNP data, it was not applied to the principal components (PCs, Figure 1E).

To rank covariate importance, we used the absolute t-statistic (penalized regression), out-of-bag mean squared error (random forest), and *P*-value (GWAS). The top nine covariates were selected using a top-M approach (M = number of true covariates).^8, 9^ For PCA, the top nine PCs were retained, sufficient to capture all nine true SNPs if each PC reflected a unique signal. From each PC, the nine SNPs with the highest loadings (81 total) were reanalyzed and re-ranked to identify the final top nine SNPs. Because a proxy SNP may be selected instead of the true causal variant, we conducted additional analyses that considered a true SNP correctly identified if either it or a highly correlated proxy (*r²* > 0.1 or 0.5) was selected.^9^

Model performance was evaluated using the F1 score, the harmonic mean of recall ($True Positive/(True Positive + False Negative)$ ) and precision ($True Positive/(True Positive + False Positive)$), calculated as: F1 = $(Recall \times Precision)/(Recall + Precision)$. In addition to mean F1 scores, we report the number of datasets achieving an F1 of 1, indicating perfect selection of all nine true covariates.^8, 9^ Runtime was recorded to assess computational burden.

**Supplementary Figures**

***Remaining SNPs***

*N = 1,235,843*

***Tentatively included SNPs***

*N = 24*

***Quality Controlled SNPs*** *N = 1,255,959*

***LD-based pruning (r^2^ = 0.1)***

***Low correlation:*** *3 randomly selected tagging SNPs*

(MAF: 5, 10, 20%)

*19,857* tagged SNPs

*19,857 tagged SNPs excluded*

***LD-based pruning (r^2^ = 0.5)***

*N = 1,236,099*

***Moderate correlation:*** *3 randomly selected tagging SNPs* (MAF: 5, 10, 20%)

*81* tagged SNPs

*81 tagged SNPs excluded*

***High correlation:*** *3 randomly selected tagging SNPs* (MAF: 5, 10, 20%)

169 tagged SNPs

Progressively sample random sets of SNPs with sizes 10^3^−𝑥, 10^4^−𝑥, 10^5^−𝑥, and 10^6^−𝑥, where 𝑥 = 24, the number of tentatively included SNPs.

Datasets ranging from 1,000–1,000,000 SNPs with 9 tagging SNPs

*154 tagged SNPs excluded^a^*

**Figure S1. SNP selection procedure for a single pharmacogenetic dataset.** Starting with 1,255,959 chromosome 1 SNPs that passed quality control (genotyping rate ≥95%, MAF ≥1%, and Hardy–Weinberg *p* < 1×10⁻⁶), datasets containing 1,000, 10,000, 100,000, and 1,000,000 SNPs were generated. Nine SNPs were selected based on their MAF (5%, 10%, 20%) and their correlations: low correlation (no SNPs with *r^2^* > 0.1 based on LD), medium (no SNPs with *r^2^* > 0.5), and high (SNPs with *r^2^* > 0.5). These nine SNPs were identical across all dataset sizes. The procedure was repeated 100 times, generating random variations in SNP composition across datasets. ^a^In this dataset, out of the 169 tagged SNPs (high correlation step), 154 were excluded, leaving 15 SNPs included. This ensured that each of the 3 tagging SNPs had exactly 5 tagged SNPs. The number 5 was set as the minimum for one of the tagging SNPs, to prevent any tag SNP from having an excessive number of tags (155) compared to the others, which had 5 and 9 tags, respectively. LD = linkage disequilibrium; MAF = minor allele frequency; SNP = single nucleotide polymorphism.

**A. Density plots**

**
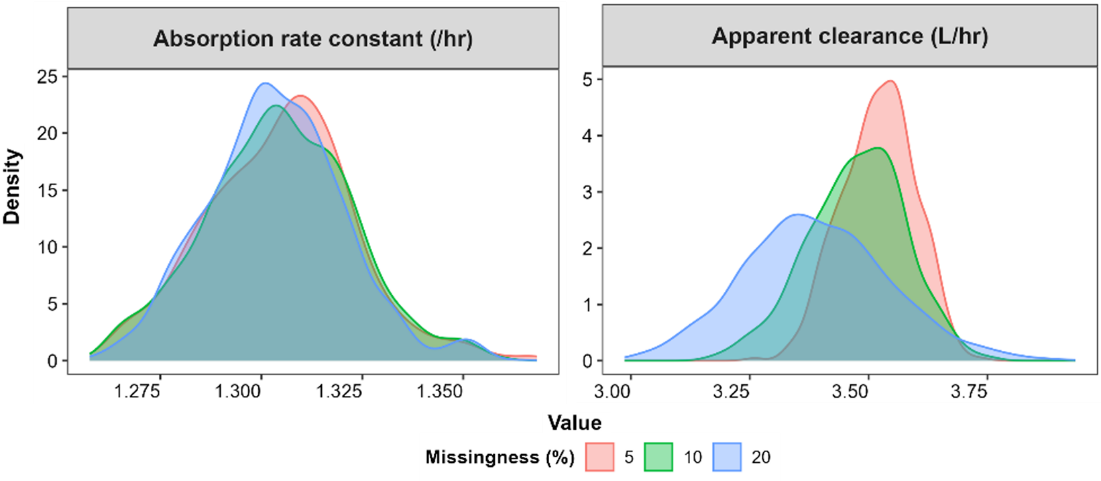
**

**B. Q-Q plots**

**
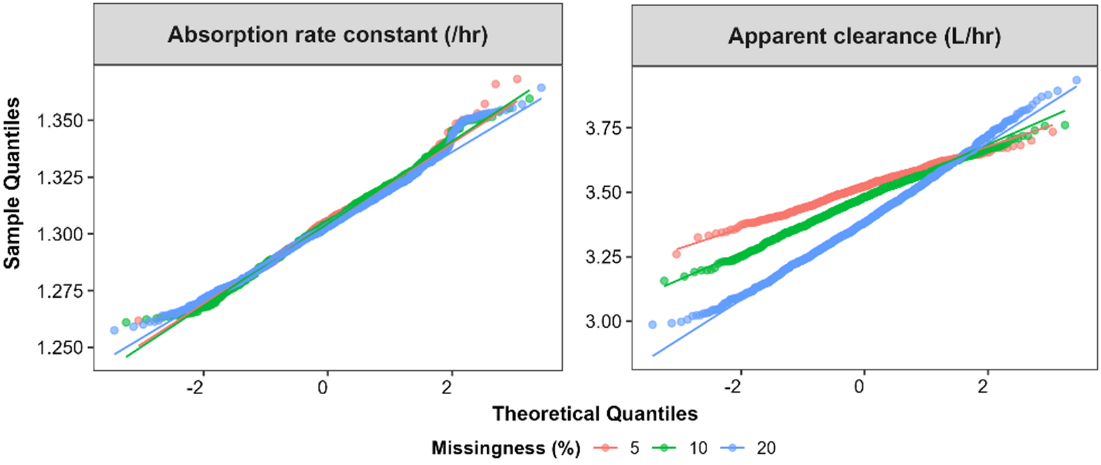
**

**Figure S2. Density (A) and Q–Q (B) plots show that imputed parameter estimates approximate a normal distribution**. Multiple imputations used predictive mean matching (100 datasets each), with the number of imputations matching the missingness level (5%, 10%, 20%), corresponding to 500, 1000, and 2000 imputations, respectively. Q-Q = quantile-quantile.


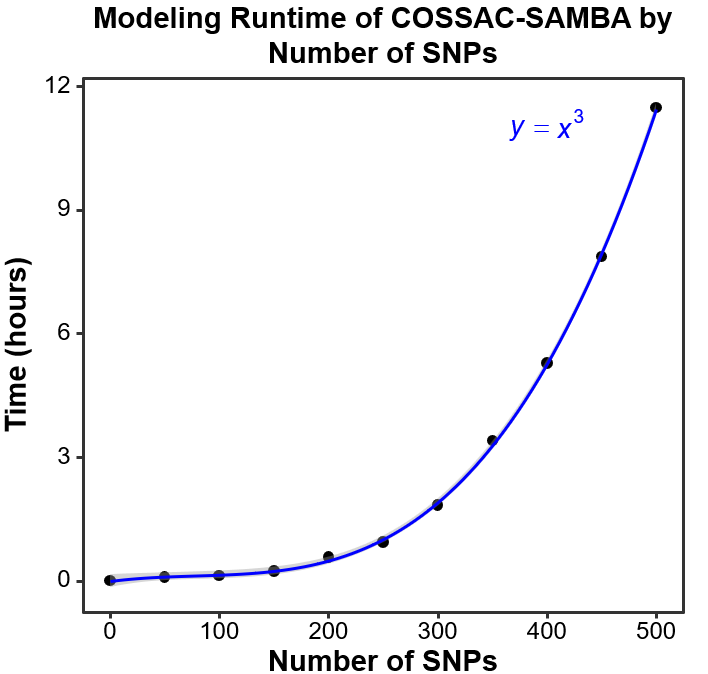


**Figure S3. Modelling runtime of Monolix’s COSSAC-SAMBA by the number of SNPs.** COSSAC **=** COnditional Sampling use for Stepwise Approach based on Correlation, SAMBA = Stochastic Approximation for Model Building Algorithm.

.

**
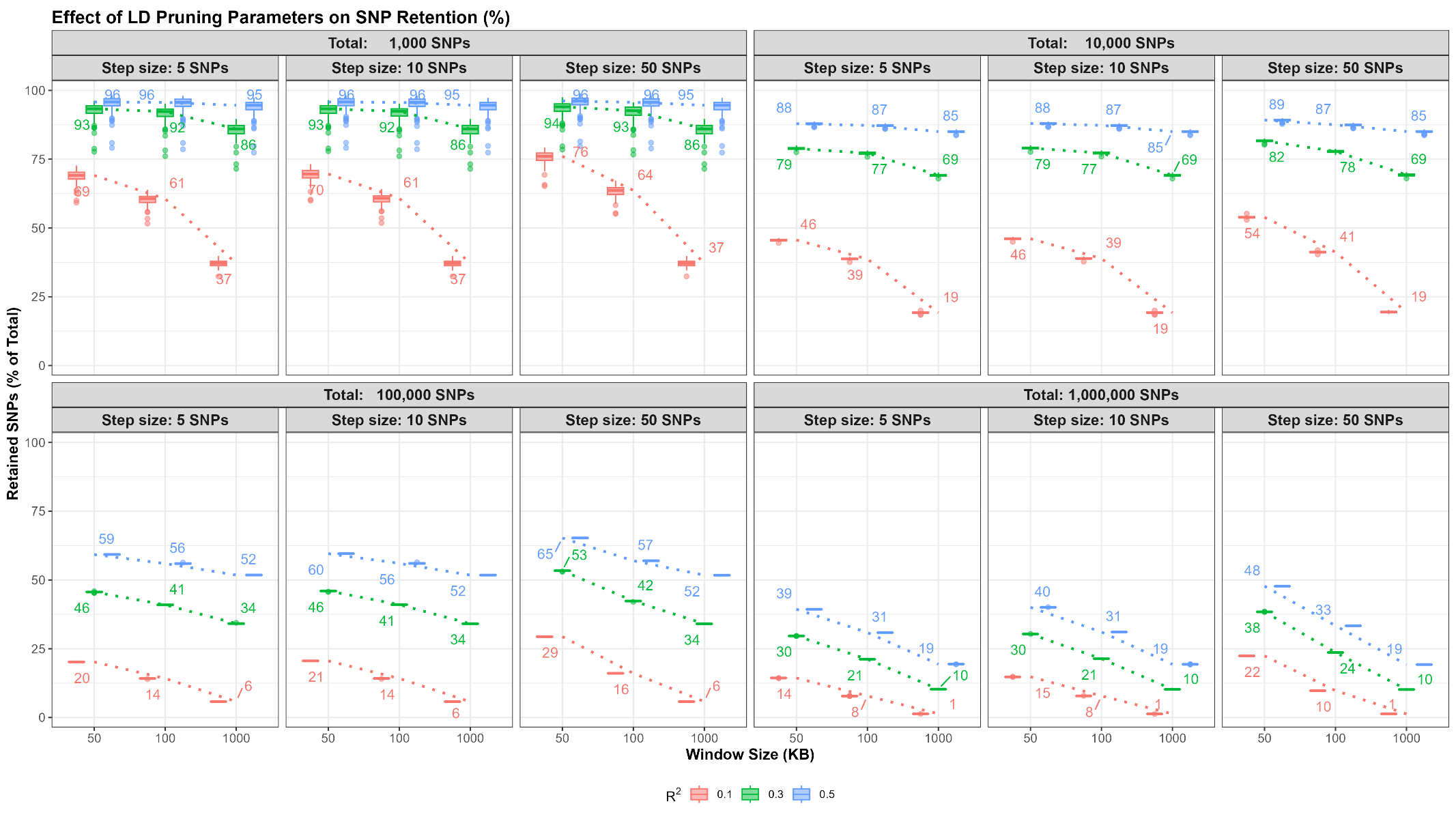
**

**Figure S4. Effect of linkage disequilibrium (LD) pruning parameters on SNP retention.** A larger window size (1,000 kb) combined with a lower *R²* threshold (0.1) resulted in the greatest SNP reduction. For instance, pruning 1,000,000 SNPs under these settings retained only ~1% (100,000 SNPs). Step size had minimal impact on retention; the default (5 SNPs) was used to maintain granularity. SNP = single nucleotide polymorphism.

**
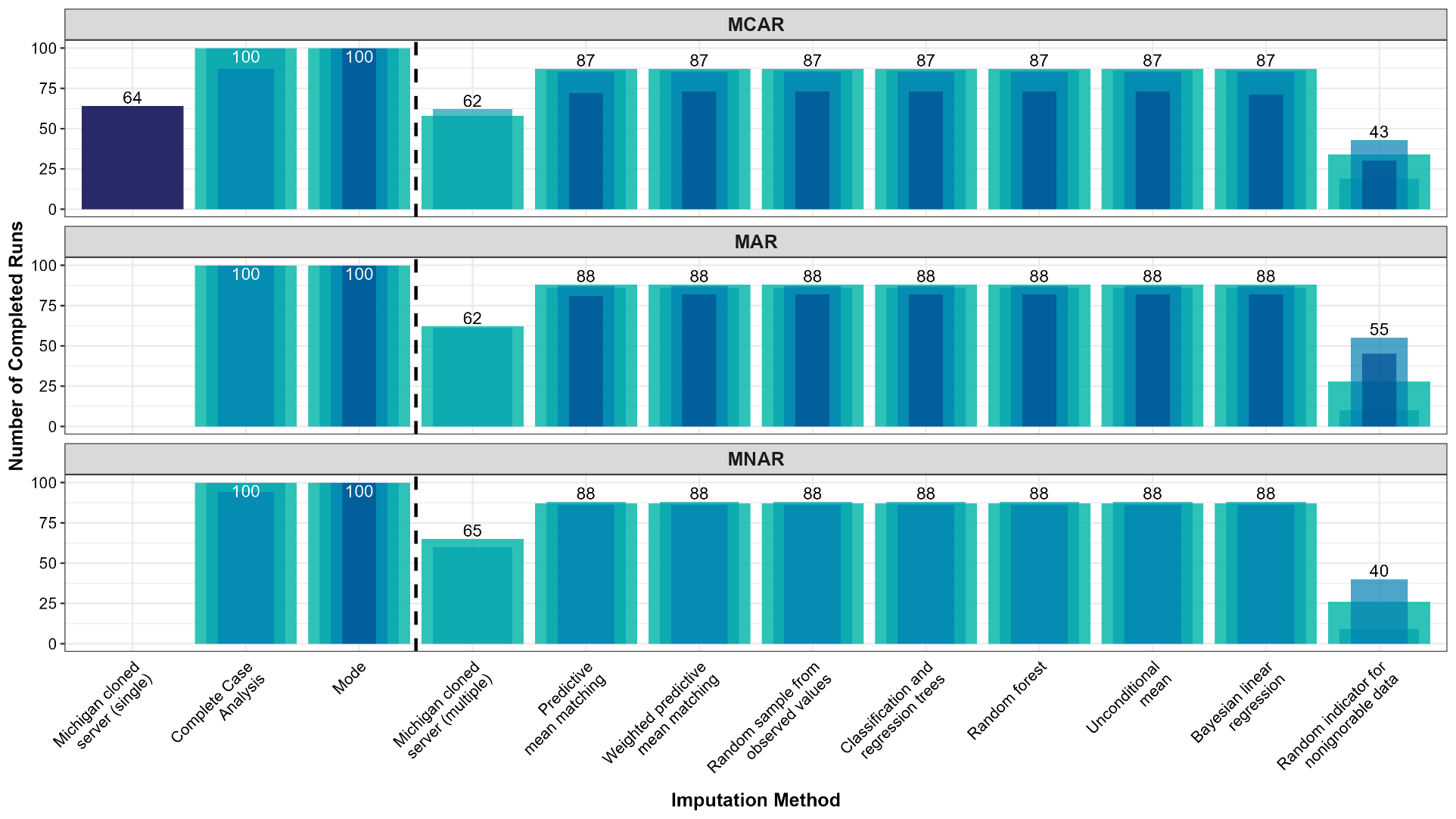
**

**Figure S5.** **Number of completed model runs across imputation methods, missing data mechanisms, and missingness levels.** Bars represent the number of successful model runs (out of 100 per effect size scenario) for each imputation method under three missingness mechanisms: missing completely at random (MCAR), missing at random (MAR), and missing not at random (MNAR). Bar colors indicate the percentage of missing SNP data (5% to 100%). The vertical dashed line separates single from multiple imputation methods. Shorter bars reflect higher failure rates. The greatest number of failed runs occurred under MNAR with 50% missingness, especially for the random indicator for nonignorable data method. The Michigan server (single imputation) is shown only under MCAR, as all SNPs were excluded, rendering missingness mechanism inapplicable. Numbers above bars indicate the highest number of completed runs per method.

**A. Low Effect Size**

**
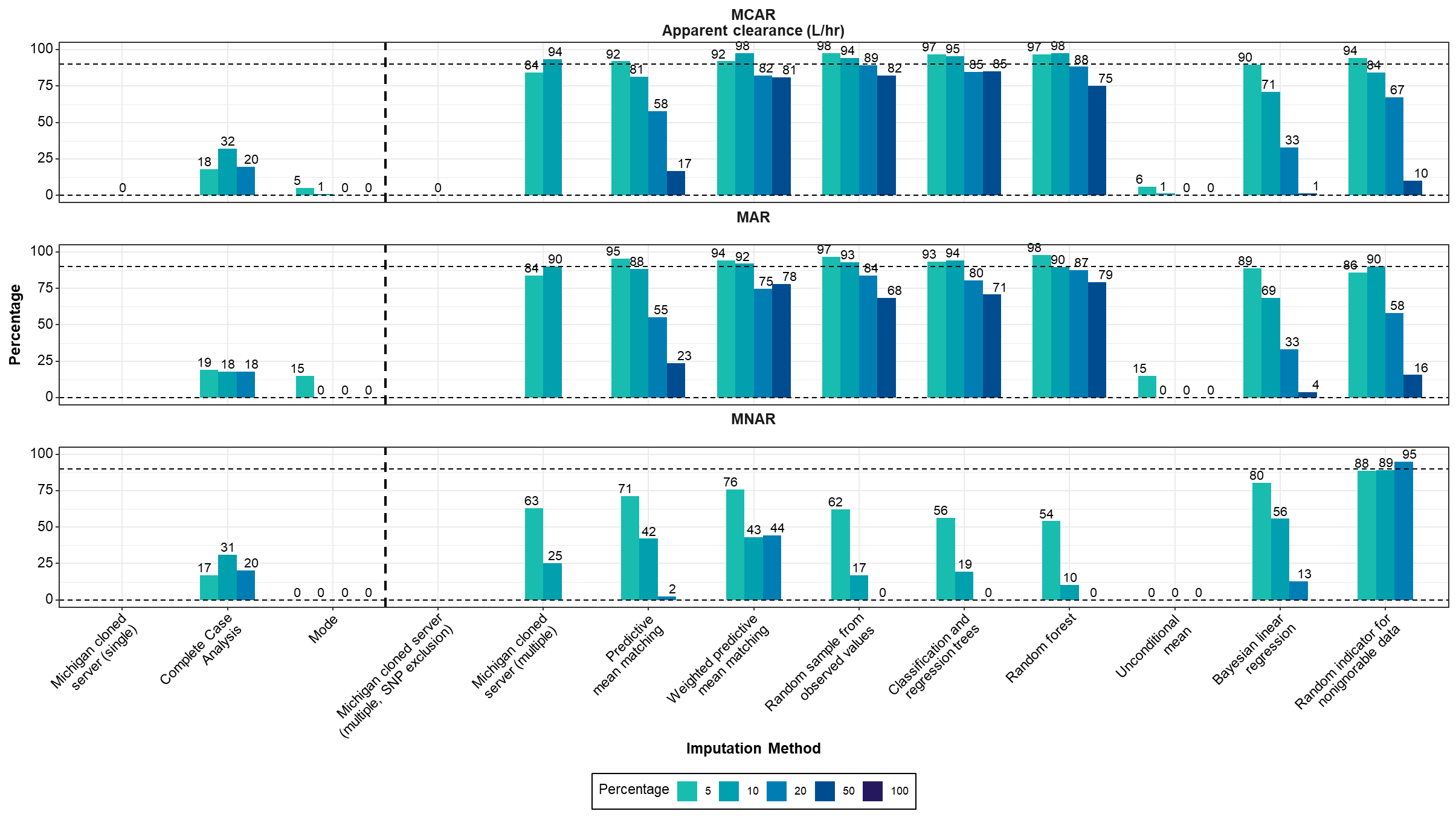
**

**
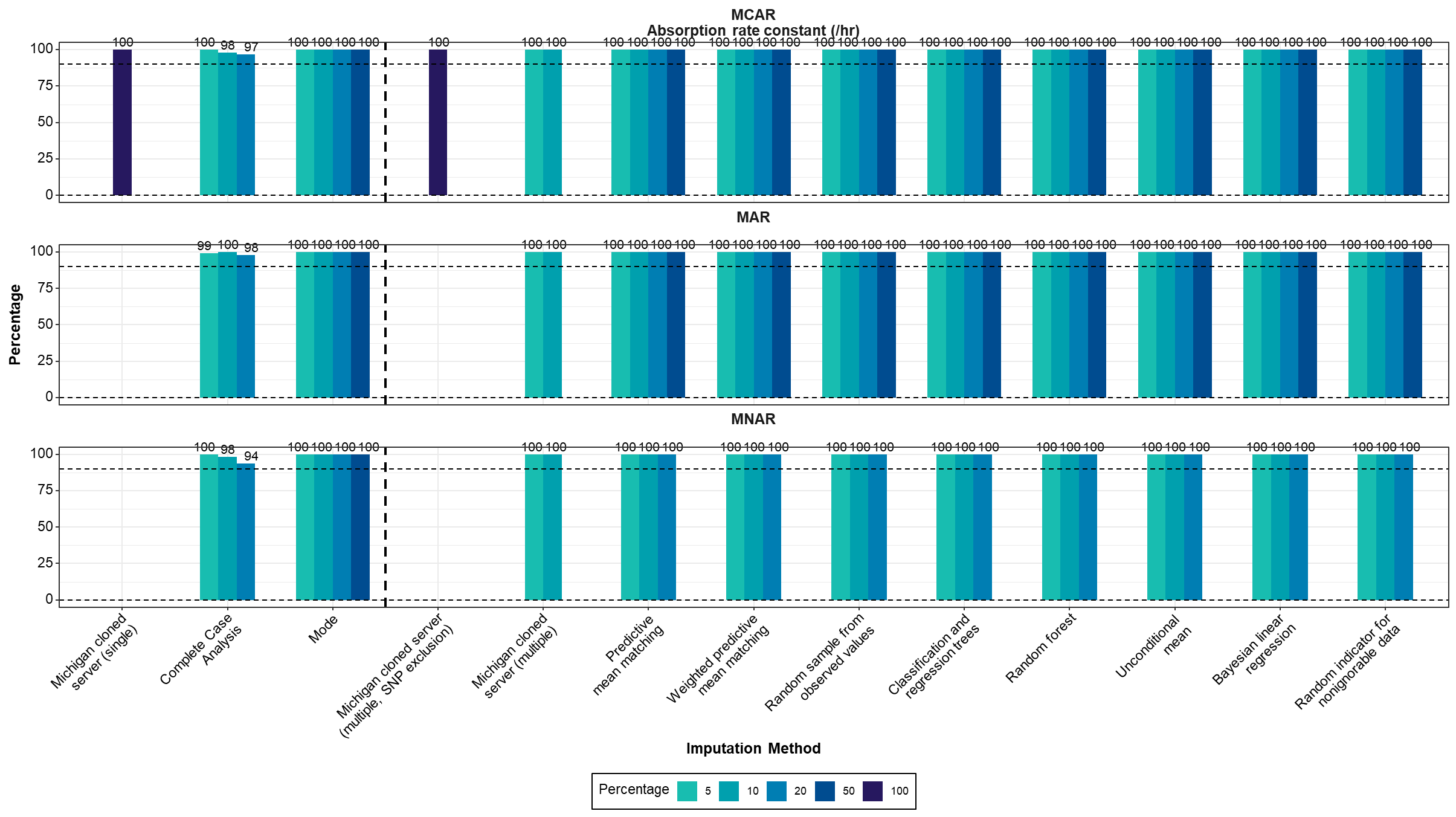
**

**B. High Effect Size**

**
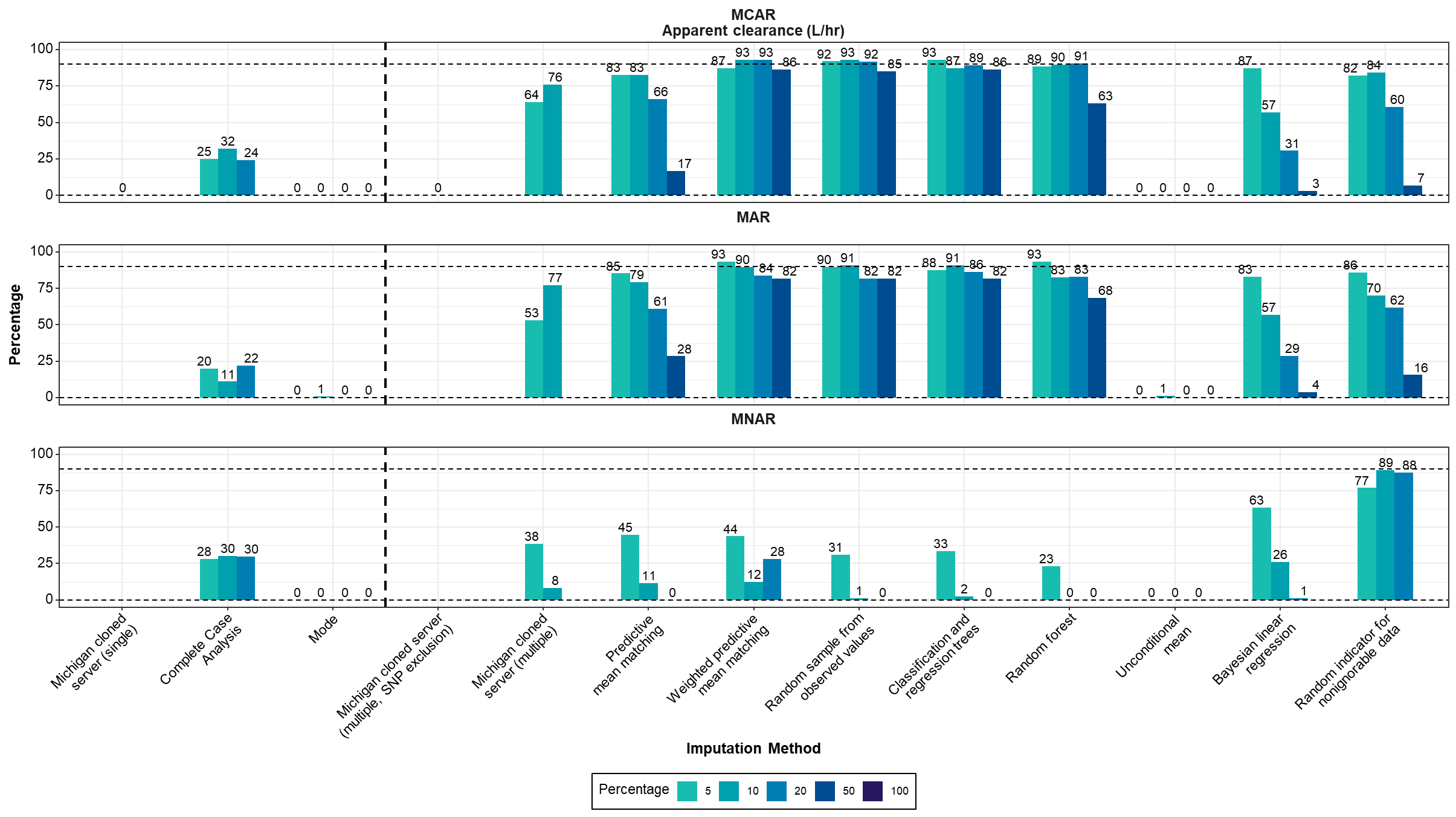
**

**
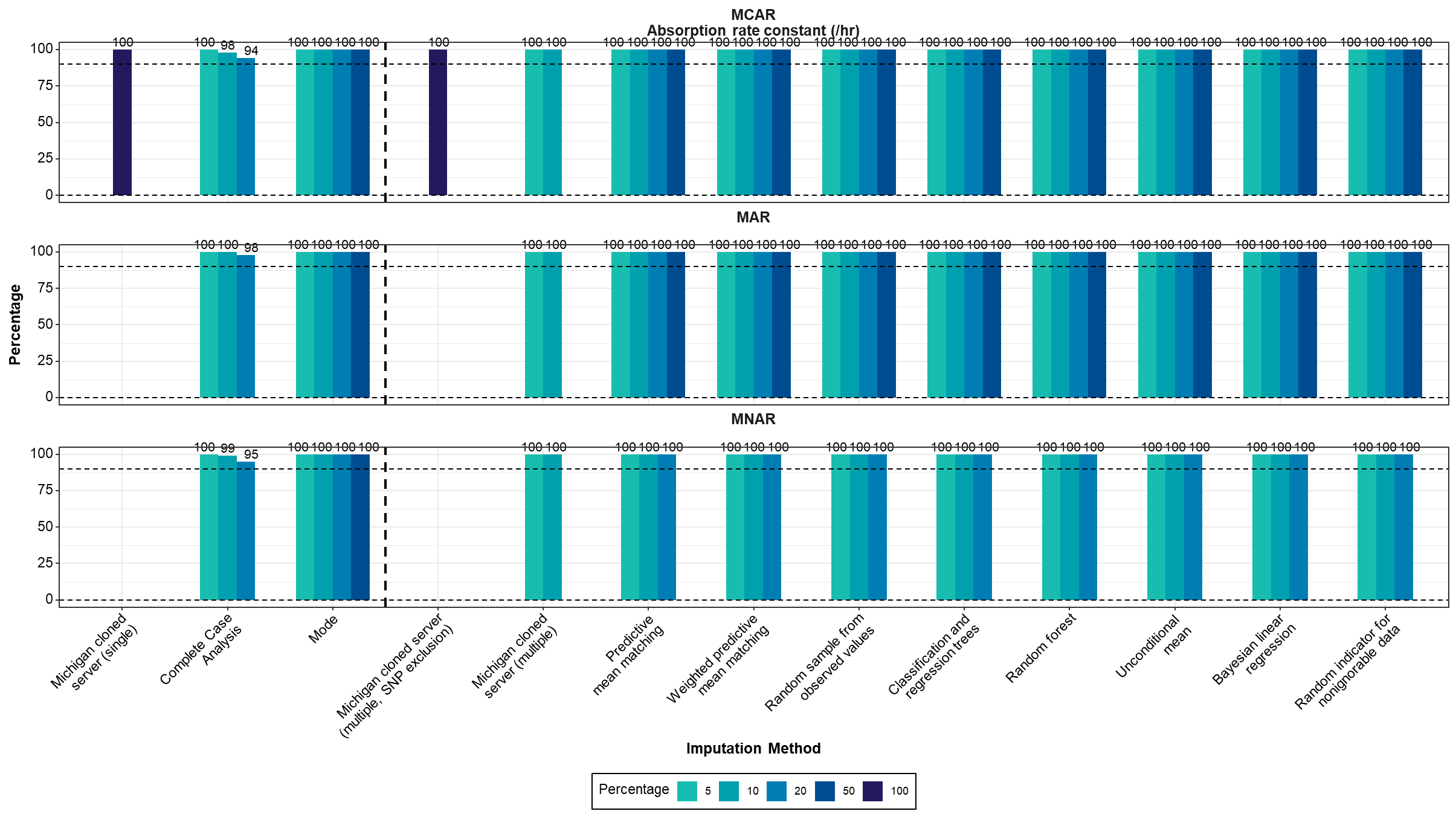
**

**Figure S6. Coverage rates of parameter estimates across imputation methods, missing data mechanisms, and effect sizes.** Panels show results for (**A**) low and (**B**) high effect size scenarios, each stratified by missingness mechanism: missing completely at random (MCAR), missing at random (MAR), and missing not at random (MNAR). Bars represent different imputation methods, with colors indicating the percentage of missing SNP data (5% to 100%). The vertical dashed line separates single from multiple imputation methods. Numbers above the bars indicate the performance; absence of a number indicates that the method was not evaluated under that condition. For example, the Michigan server (single imputation) excluded SNPs with high missingness (i.e. 100% missingness for those SNPs) and was therefore evaluated only once. It is displayed only under MCAR, as SNP exclusion renders the missingness mechanism inapplicable. In contrast, the multiple imputation strategy using the Michigan server was assessed at two missingness levels (5% and 10%). MICE = multivariate imputation by chained equations; SNP = single nucleotide polymorphism.

**A. Low Effect Size
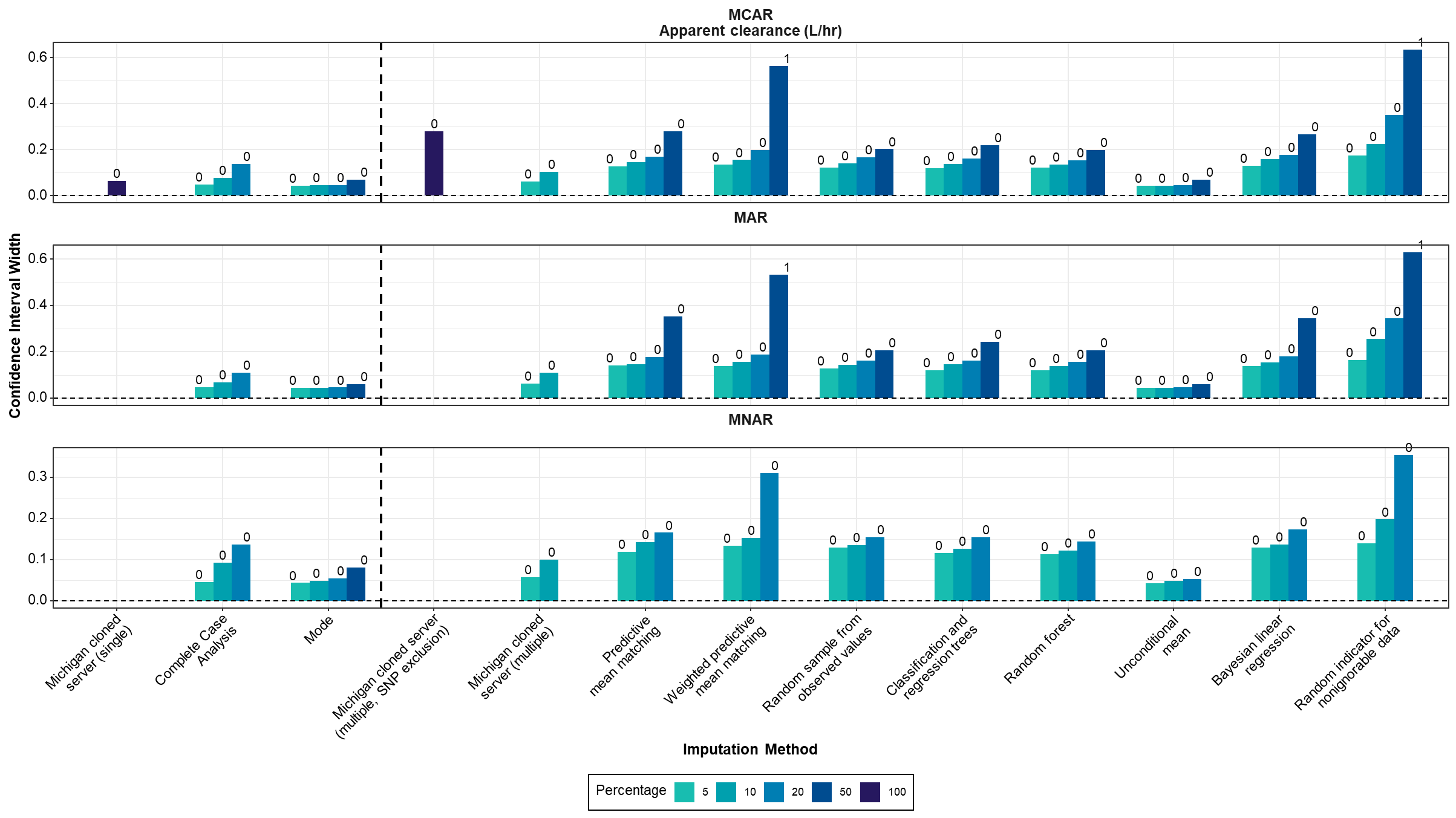
**

**B. High Effect Size**

**
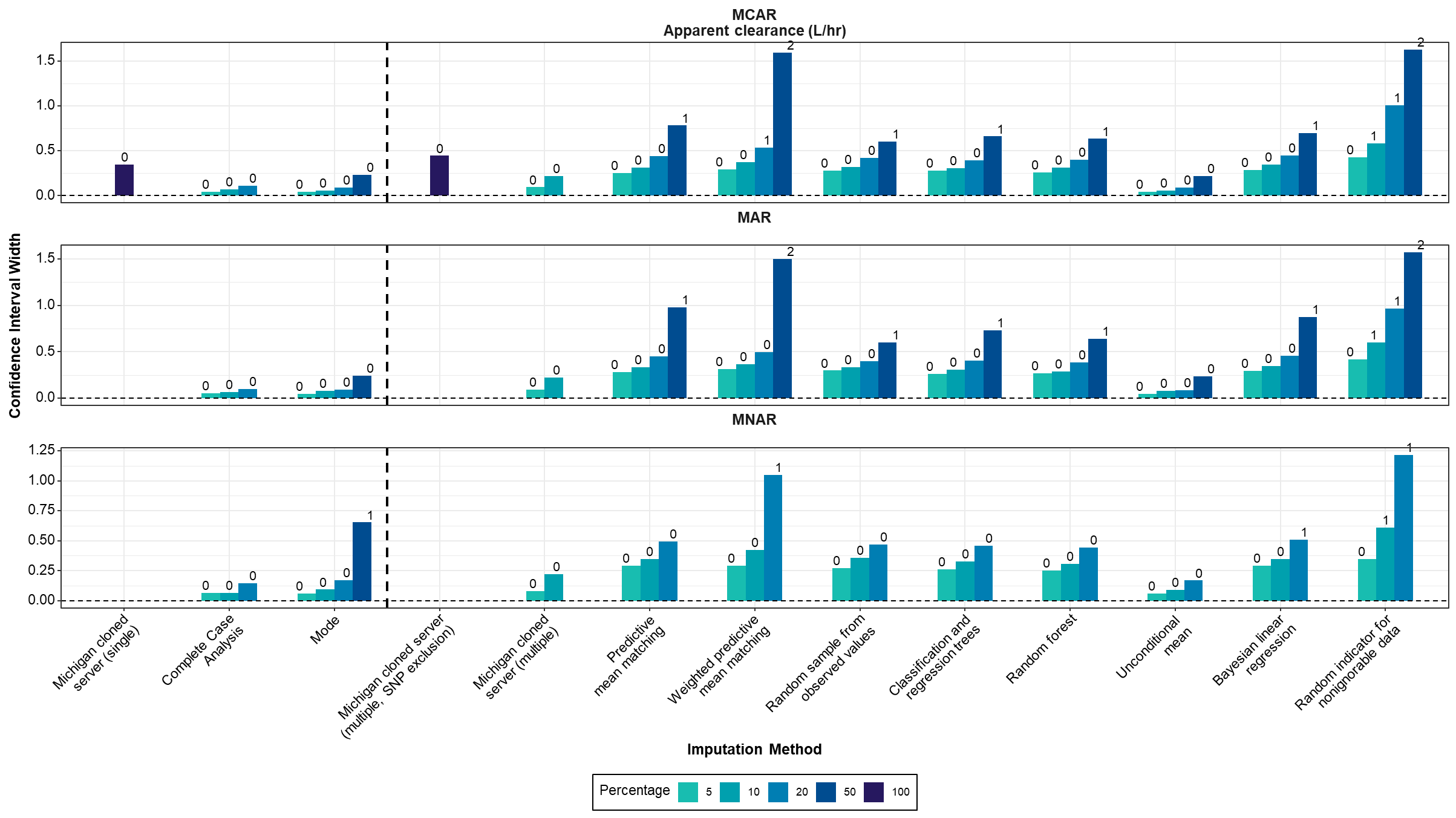
**

**Figure S7. Average Confidence Interval Width of apparent clearance estimates across imputation methods, missing data mechanisms, and effect sizes.** Panels show results for (**A**) low and (**B**) high effect size scenarios, each stratified by missingness mechanism: missing completely at random (MCAR), missing at random (MAR), and missing not at random (MNAR). Bars represent different imputation methods, with colors indicating the percentage of missing SNP data (5% to 100%). The vertical dashed line separates single from multiple imputation methods. Numbers above the bars indicate the performance; absence of a number indicates that the method was not evaluated under that condition. For example, the Michigan server (single imputation) excluded SNPs with high missingness (i.e. 100% missingness for those SNPs) and was therefore evaluated only once. It is displayed only under MCAR, as SNP exclusion renders the missingness mechanism inapplicable. In contrast, the multiple imputation strategy using the Michigan server was assessed at two missingness levels (5% and 10%). MICE = multivariate imputation by chained equations; SNP = single nucleotide polymorphism.

**A. Low Effect Size
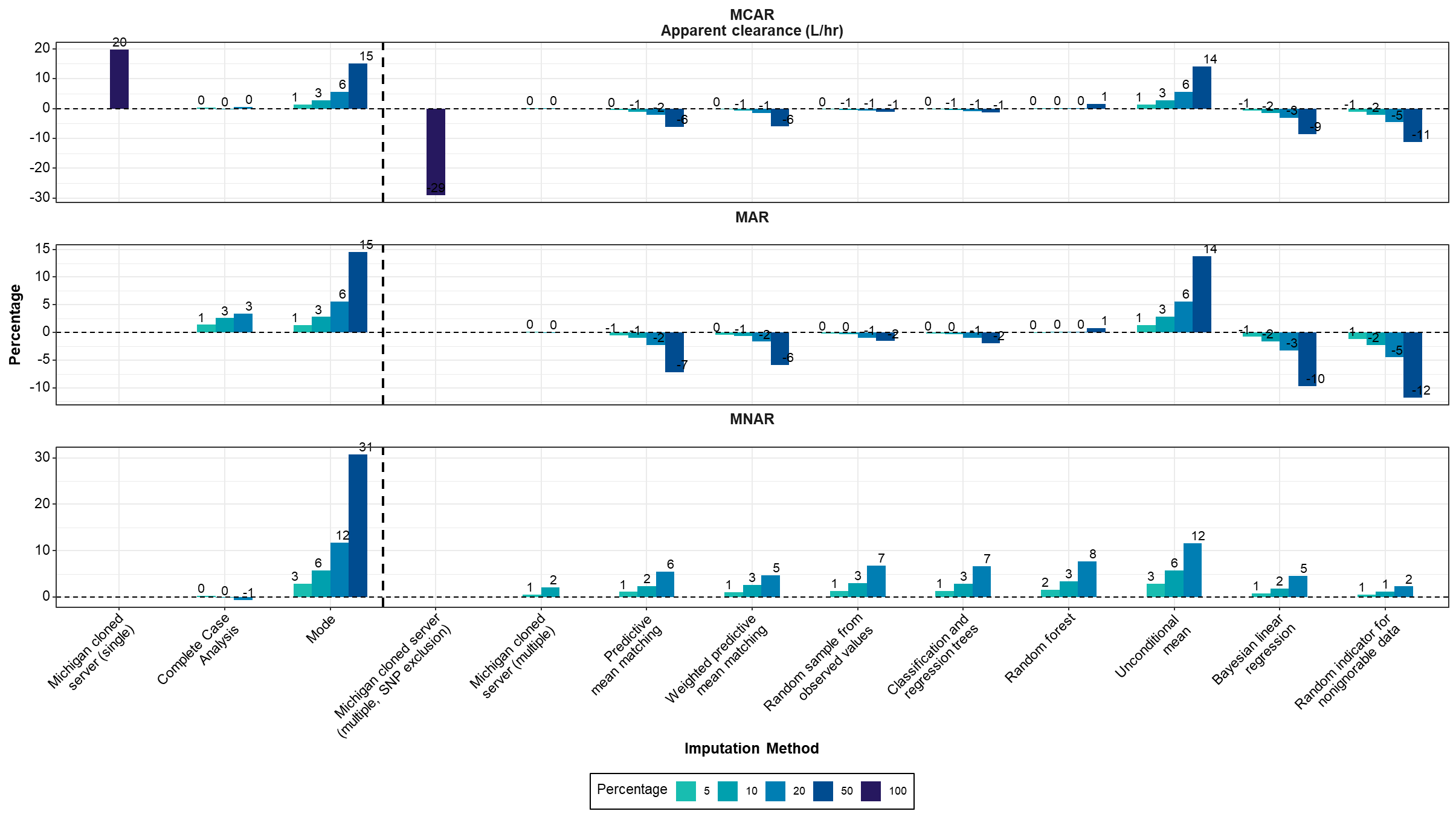
**

**B. High Effect Size**

**
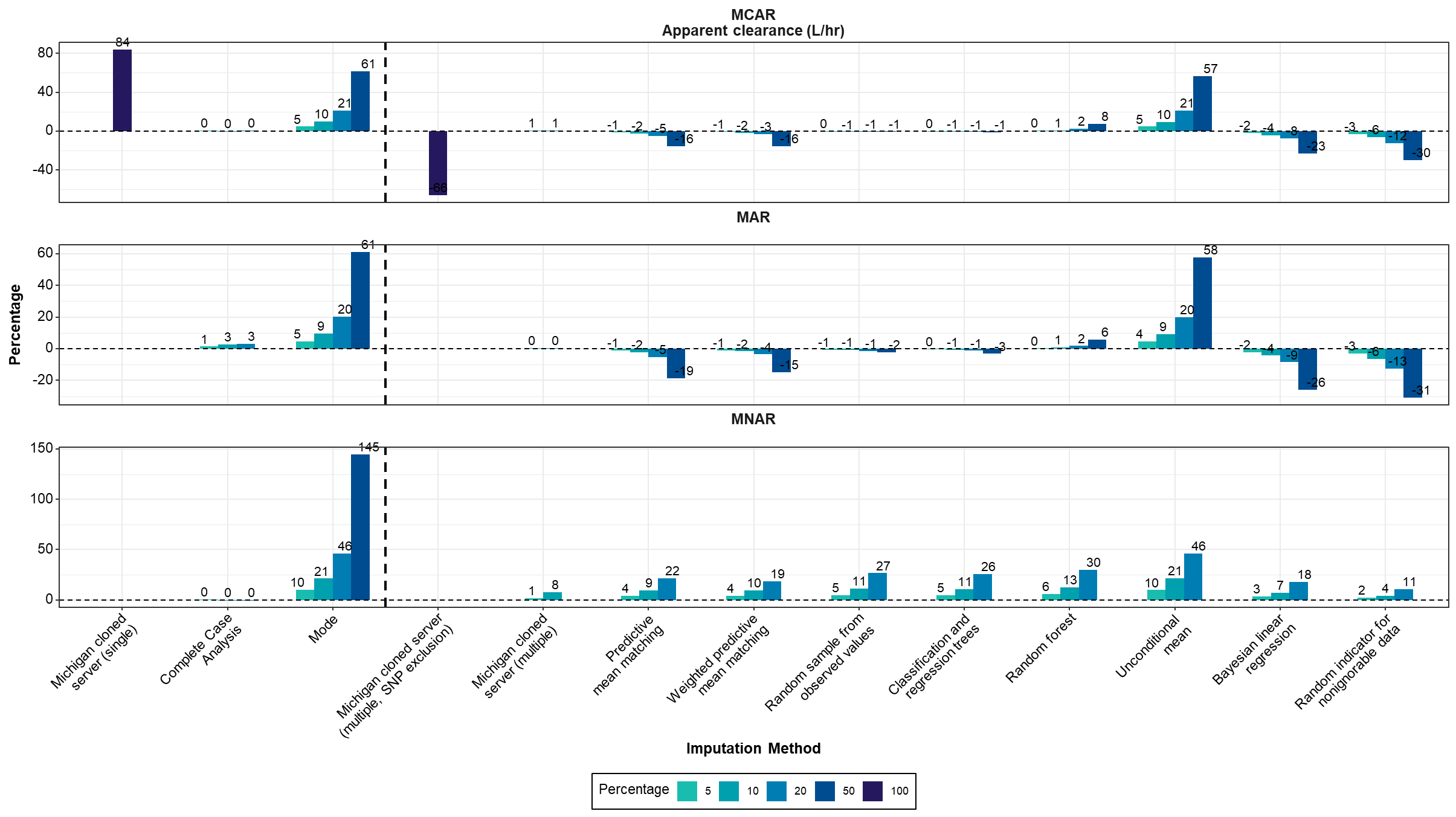
**

**Figure S8. Exponent of the logarithm of the accuracy ratio (eMLAR) of apparent clearance estimates across imputation methods, missing data mechanisms, and effect sizes.** Panels show results for (**A**) low and (**B**) high effect size scenarios, each stratified by missingness mechanism: missing completely at random (MCAR), missing at random (MAR), and missing not at random (MNAR). Bars represent different imputation methods, with colors indicating the percentage of missing SNP data (5% to 100%). The vertical dashed line separates single from multiple imputation methods. Numbers above the bars indicate the performance; absence of a number indicates that the method was not evaluated under that condition. For example, the Michigan server (single imputation) excluded SNPs with high missingness (i.e. 100% missingness for those SNPs) and was therefore evaluated only once. It is displayed only under MCAR, as SNP exclusion renders the missingness mechanism inapplicable. In contrast, the multiple imputation strategy using the Michigan server was assessed at two missingness levels (5% and 10%). MICE = multivariate imputation by chained equations; SNP = single nucleotide polymorphism.

**A. Low Effect Size
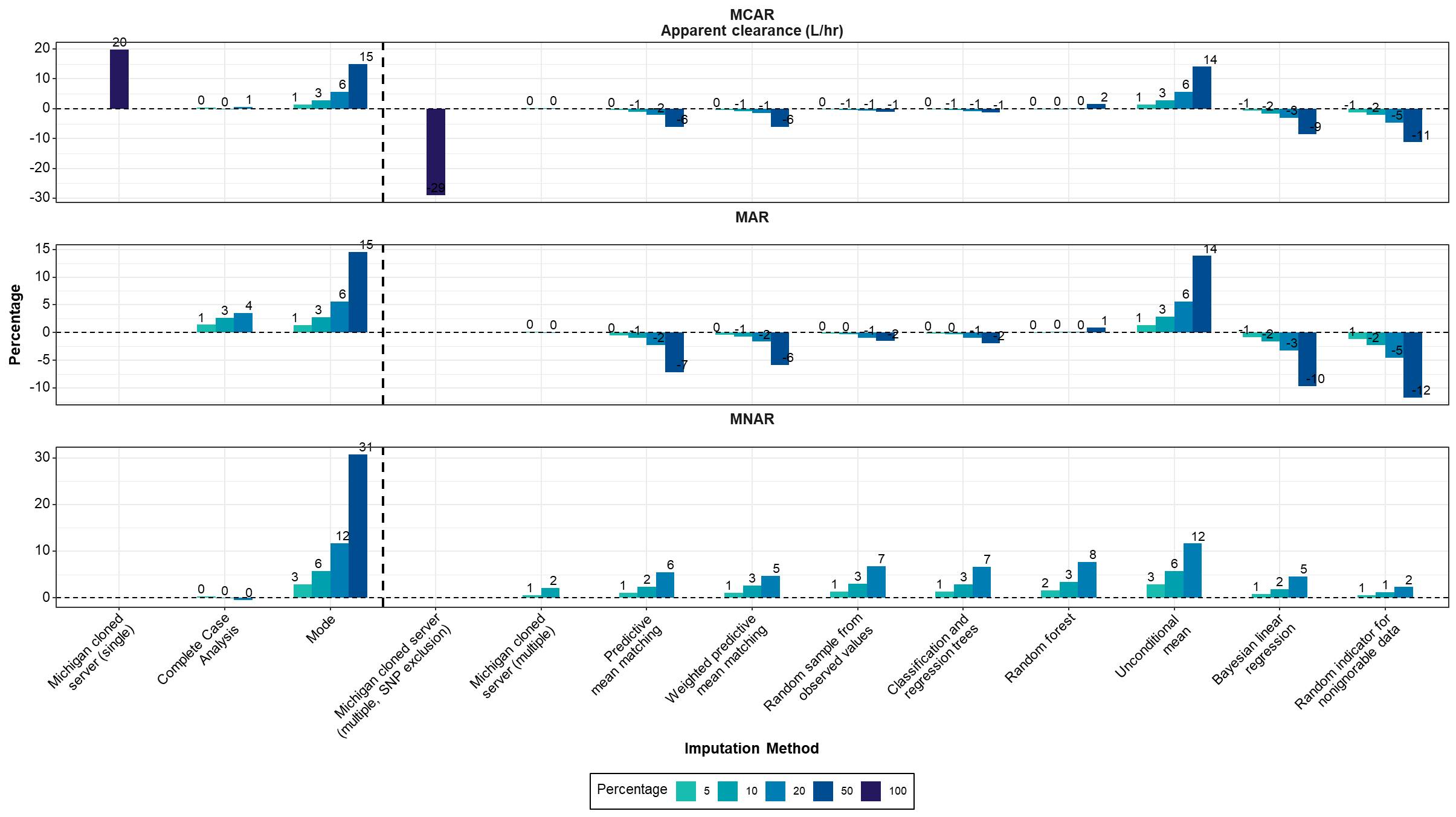
**

**B. High Effect Size**

**
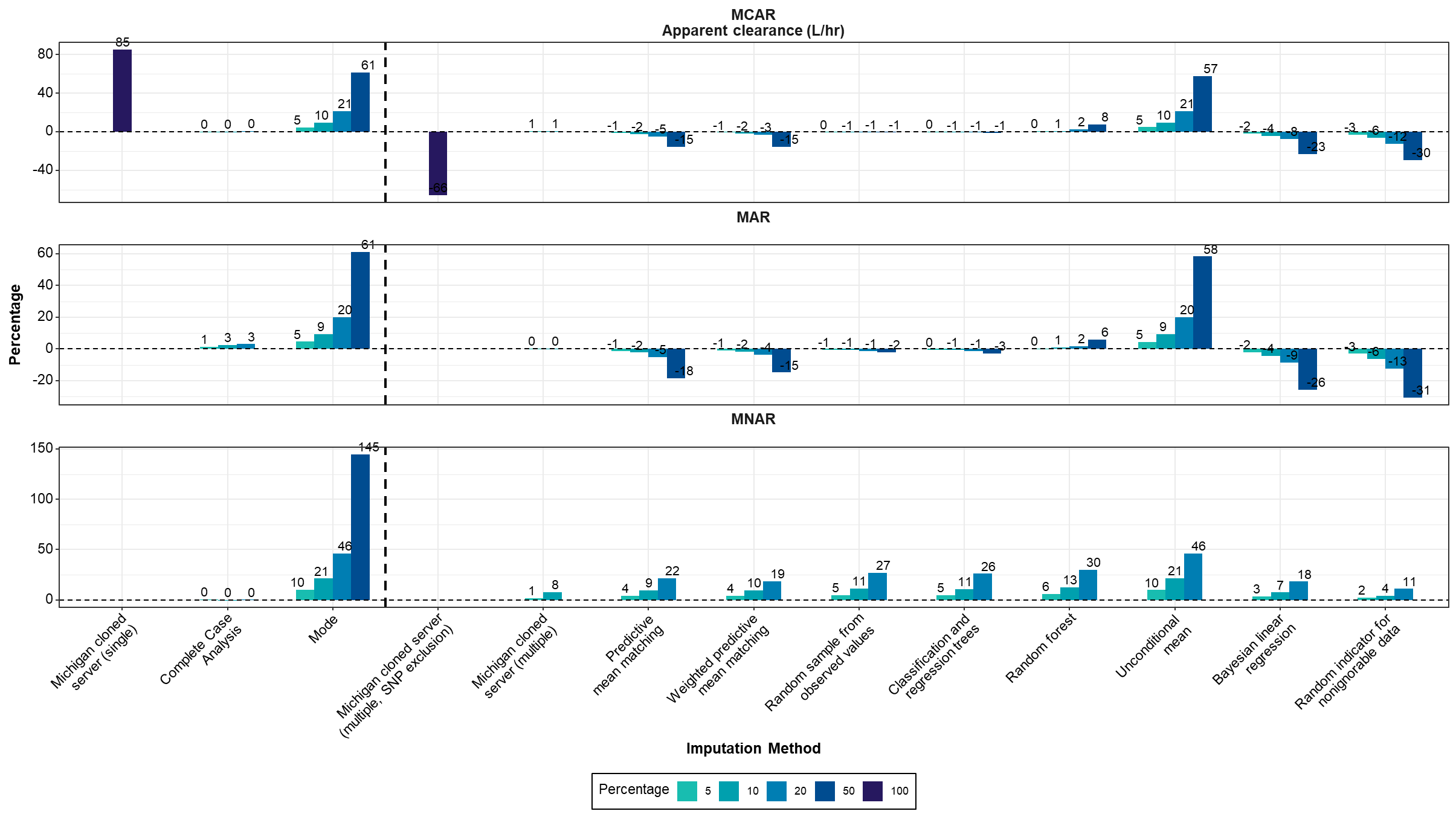
**

**Figure S9. Mean relative prediction error (MRPE) of apparent clearance estimates across imputation methods, missing data mechanisms, and effect sizes.** Panels show results for (**A**) low and (**B**) high effect size scenarios, each stratified by missingness mechanism: missing completely at random (MCAR), missing at random (MAR), and missing not at random (MNAR). Bars represent different imputation methods, with colors indicating the percentage of missing SNP data (5% to 100%). The vertical dashed line separates single from multiple imputation methods. Numbers above the bars indicate the performance; absence of a number indicates that the method was not evaluated under that condition. For example, the Michigan server (single imputation) excluded SNPs with high missingness (i.e. 100% missingness for those SNPs) and was therefore evaluated only once. It is displayed only under MCAR, as SNP exclusion renders the missingness mechanism inapplicable. In contrast, the multiple imputation strategy using the Michigan server was assessed at two missingness levels (5% and 10%). MICE = multivariate imputation by chained equations; SNP = single nucleotide polymorphism.

**A. Low Effect Size
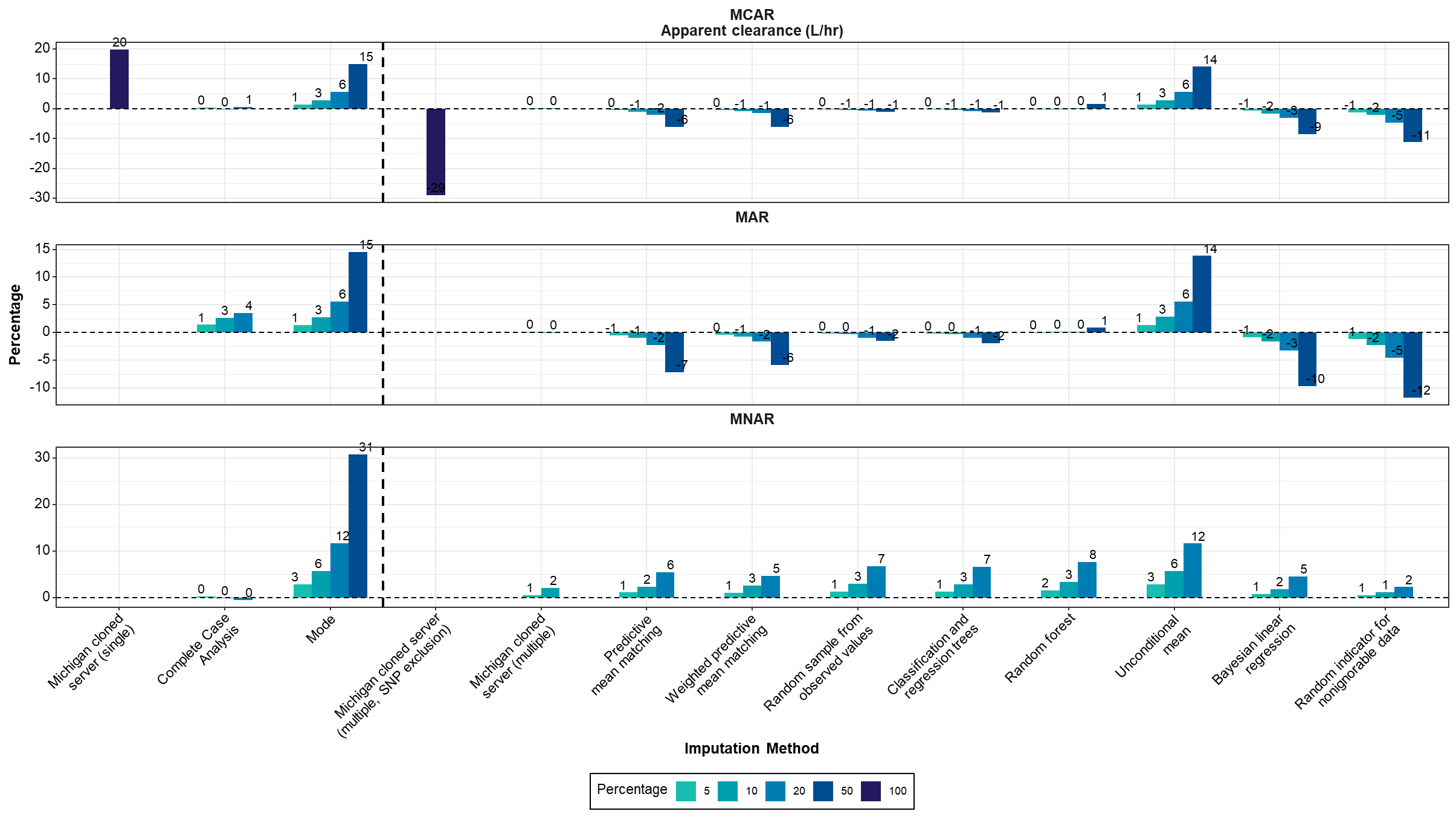
**

**B. High Effect Size**

**
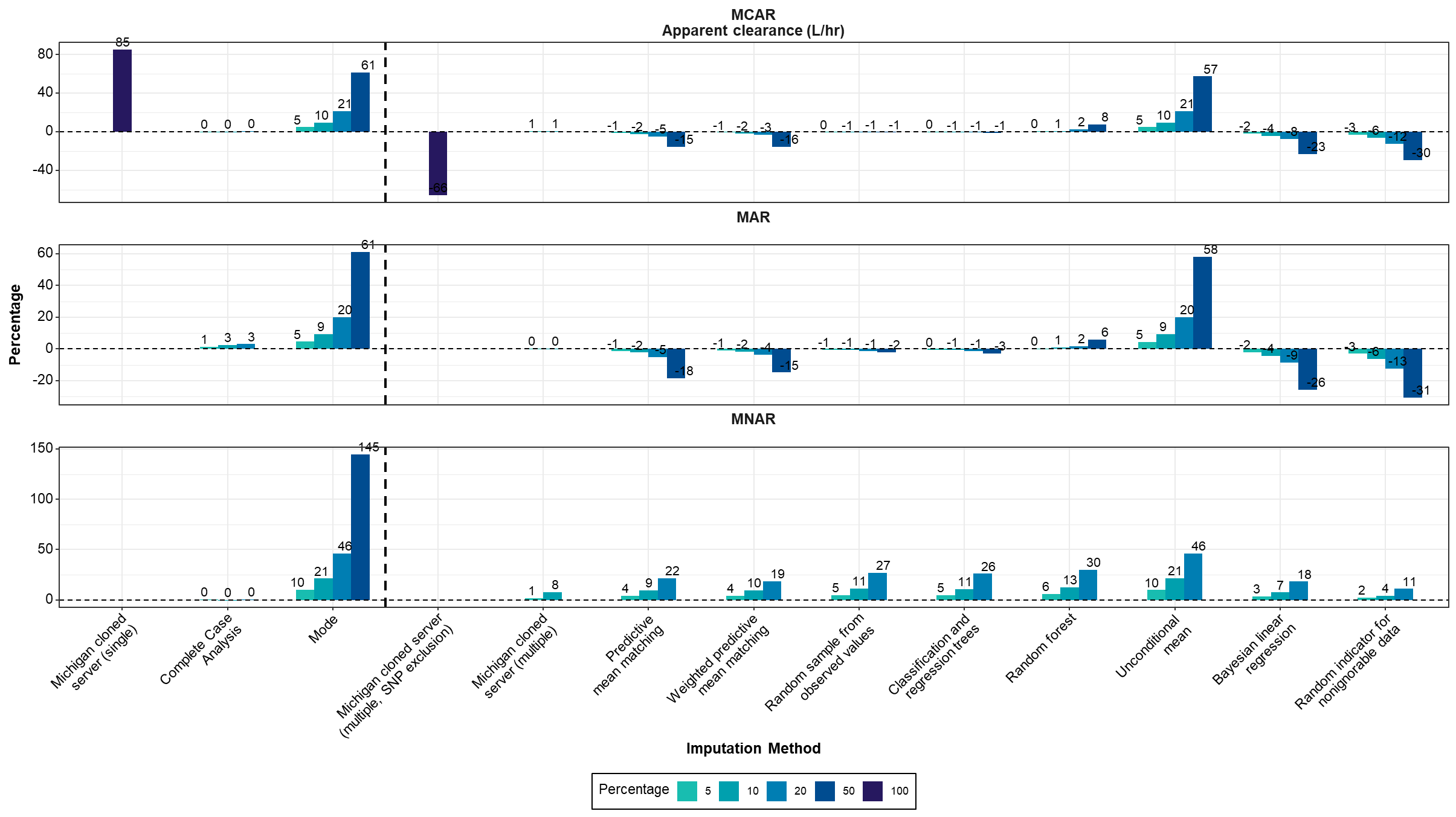
**

**Figure S10. Relative mean prediction error (rMPE) of apparent clearance estimates across imputation methods, missing data mechanisms, and effect sizes.** Panels show results for (**A**) low and (**B**) high effect size scenarios, each stratified by missingness mechanism: missing completely at random (MCAR), missing at random (MAR), and missing not at random (MNAR). Bars represent different imputation methods, with colors indicating the percentage of missing SNP data (5% to 100%). The vertical dashed line separates single from multiple imputation methods. Numbers above the bars indicate the performance; absence of a number indicates that the method was not evaluated under that condition. For example, the Michigan server (single imputation) excluded SNPs with high missingness (i.e. 100% missingness for those SNPs) and was therefore evaluated only once. It is displayed only under MCAR, as SNP exclusion renders the missingness mechanism inapplicable. In contrast, the multiple imputation strategy using the Michigan server was assessed at two missingness levels (5% and 10%). MICE = multivariate imputation by chained equations; SNP = single nucleotide polymorphism.

**A. Low Effect Size
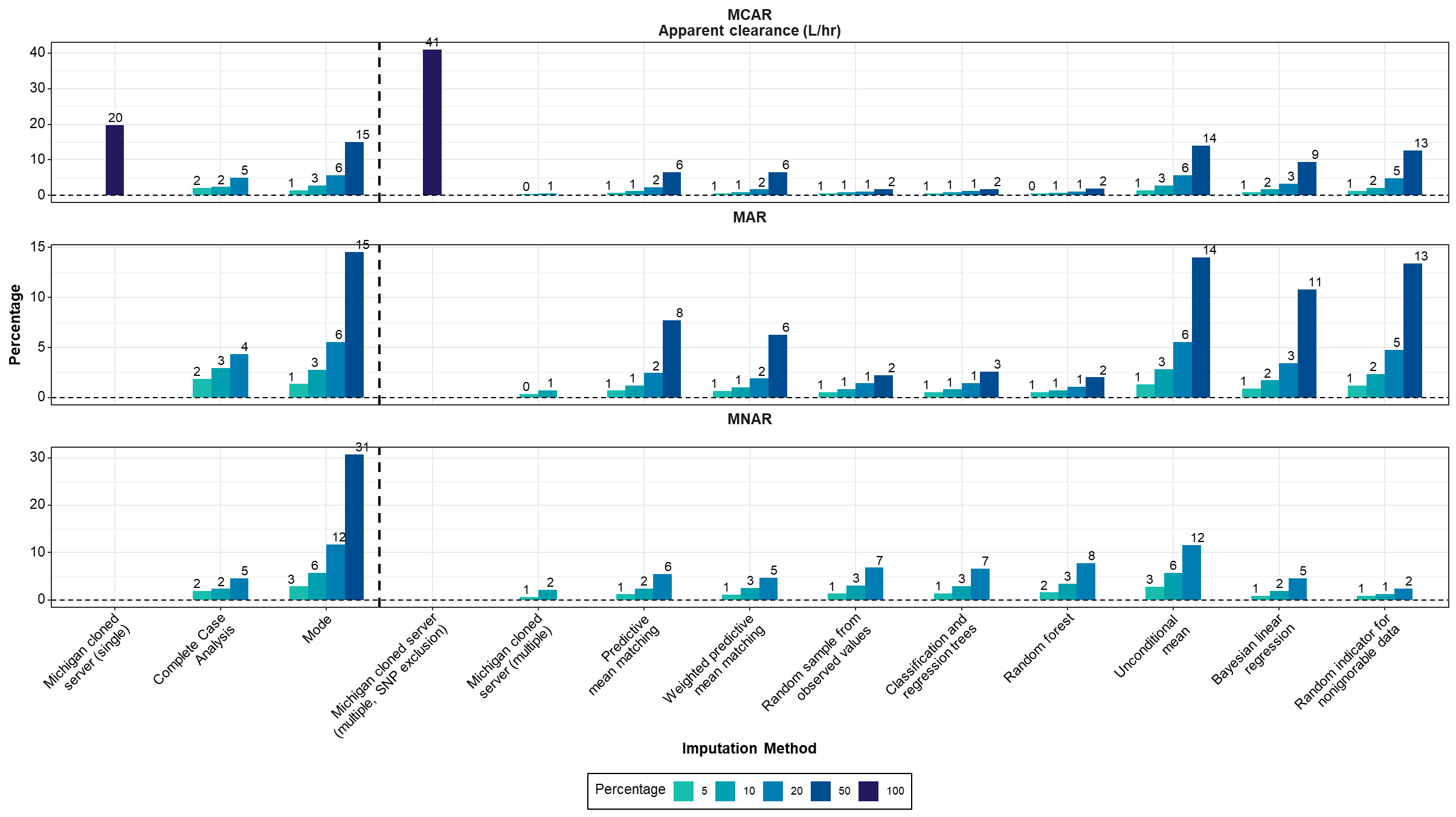
**

**B. High Effect Size**

**
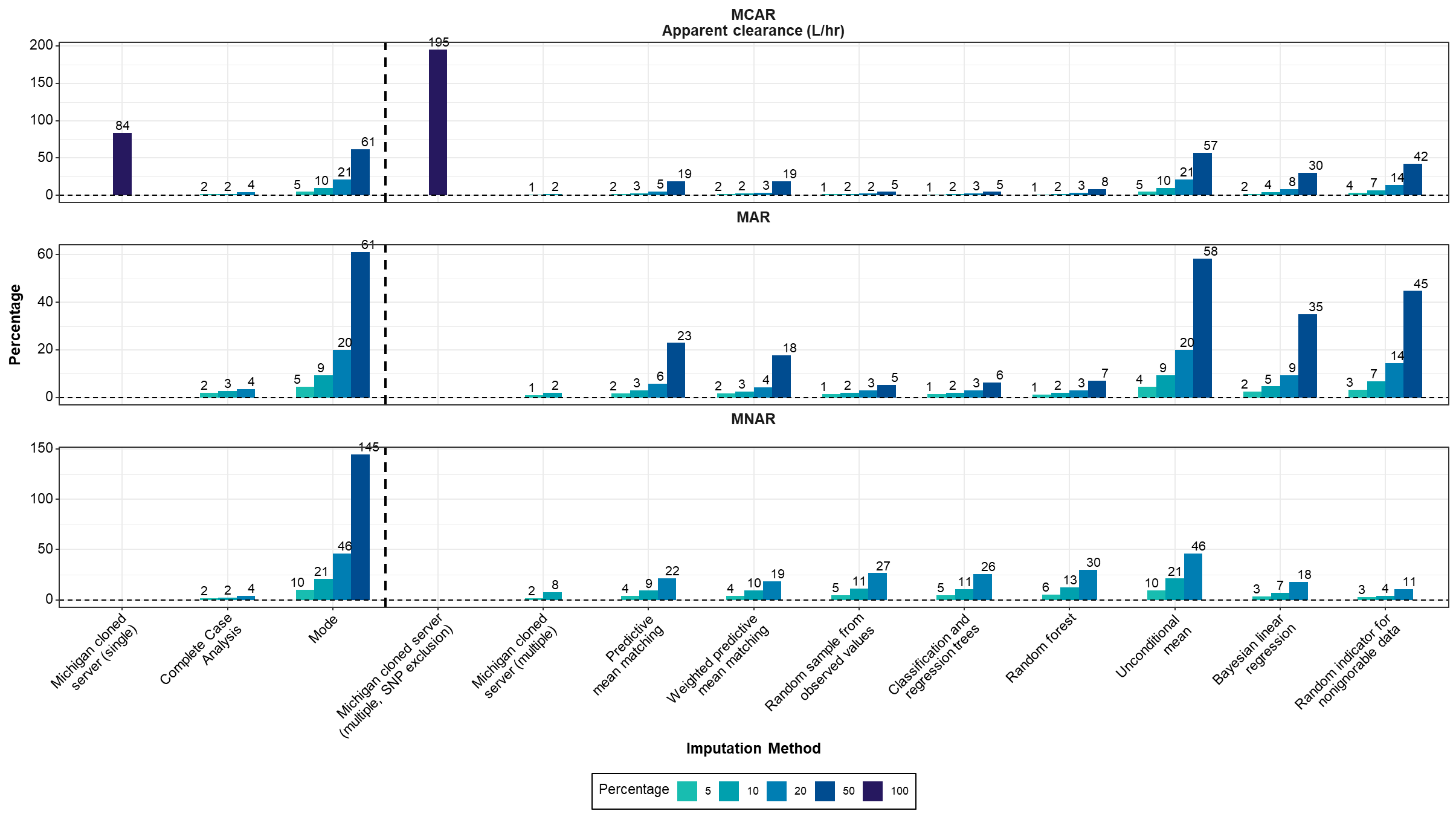
**

**Figure S11. Exponent of the absolute logarithm of the accuracy ratio (eMALAR) of apparent clearance estimates across imputation methods, missing data mechanisms, and effect sizes.** Panels show results for (**A**) low and (**B**) high effect size scenarios, each stratified by missingness mechanism: missing completely at random (MCAR), missing at random (MAR), and missing not at random (MNAR). Bars represent different imputation methods, with colors indicating the percentage of missing SNP data (5% to 100%). The vertical dashed line separates single from multiple imputation methods. Numbers above the bars indicate the performance; absence of a number indicates that the method was not evaluated under that condition. For example, the Michigan server (single imputation) excluded SNPs with high missingness (i.e. 100% missingness for those SNPs) and was therefore evaluated only once. It is displayed only under MCAR, as SNP exclusion renders the missingness mechanism inapplicable. In contrast, the multiple imputation strategy using the Michigan server was assessed at two missingness levels (5% and 10%). MICE = multivariate imputation by chained equations; SNP = single nucleotide polymorphism.

**A. Low Effect Size
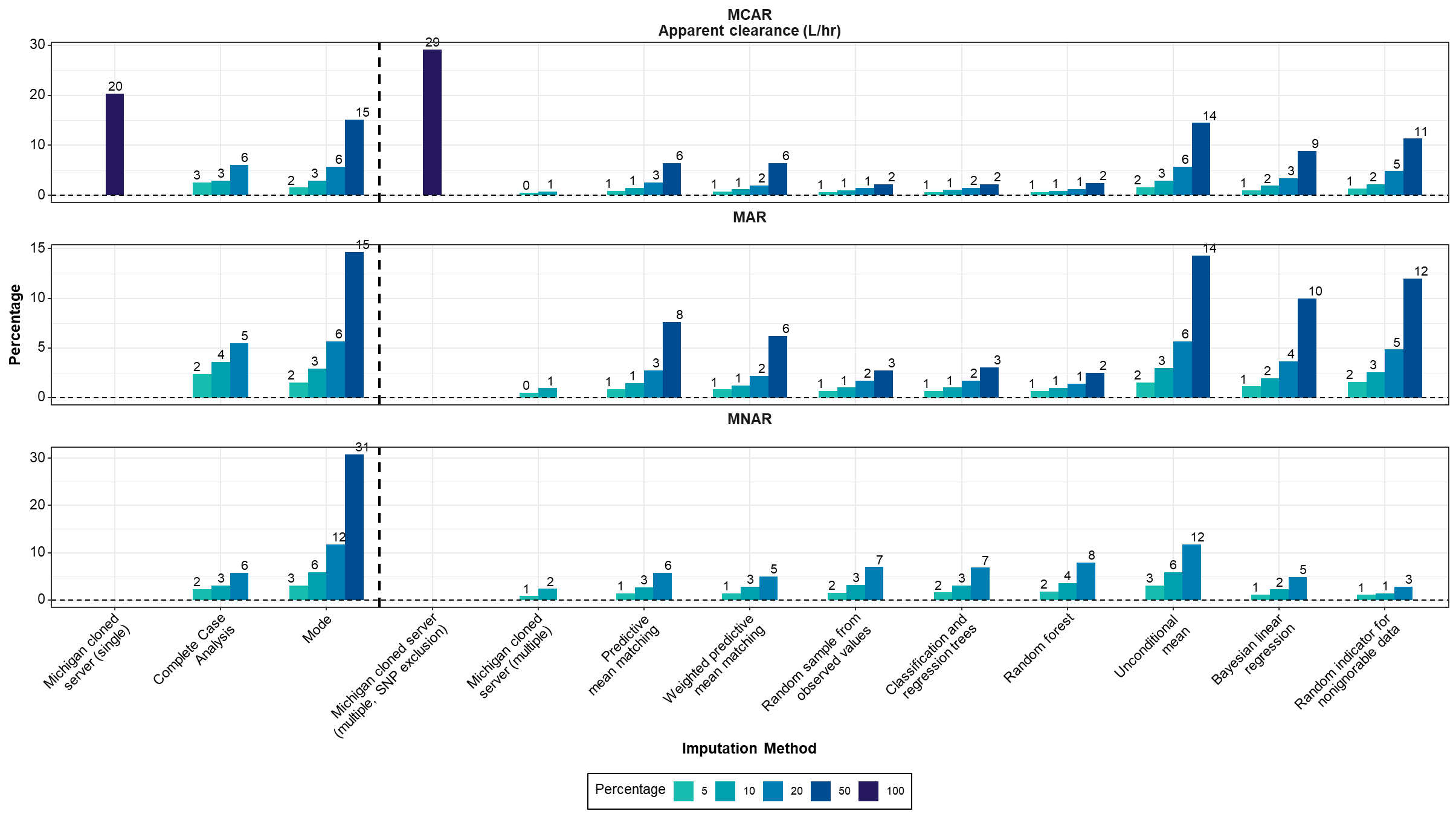
**

**B. High Effect Size**

**
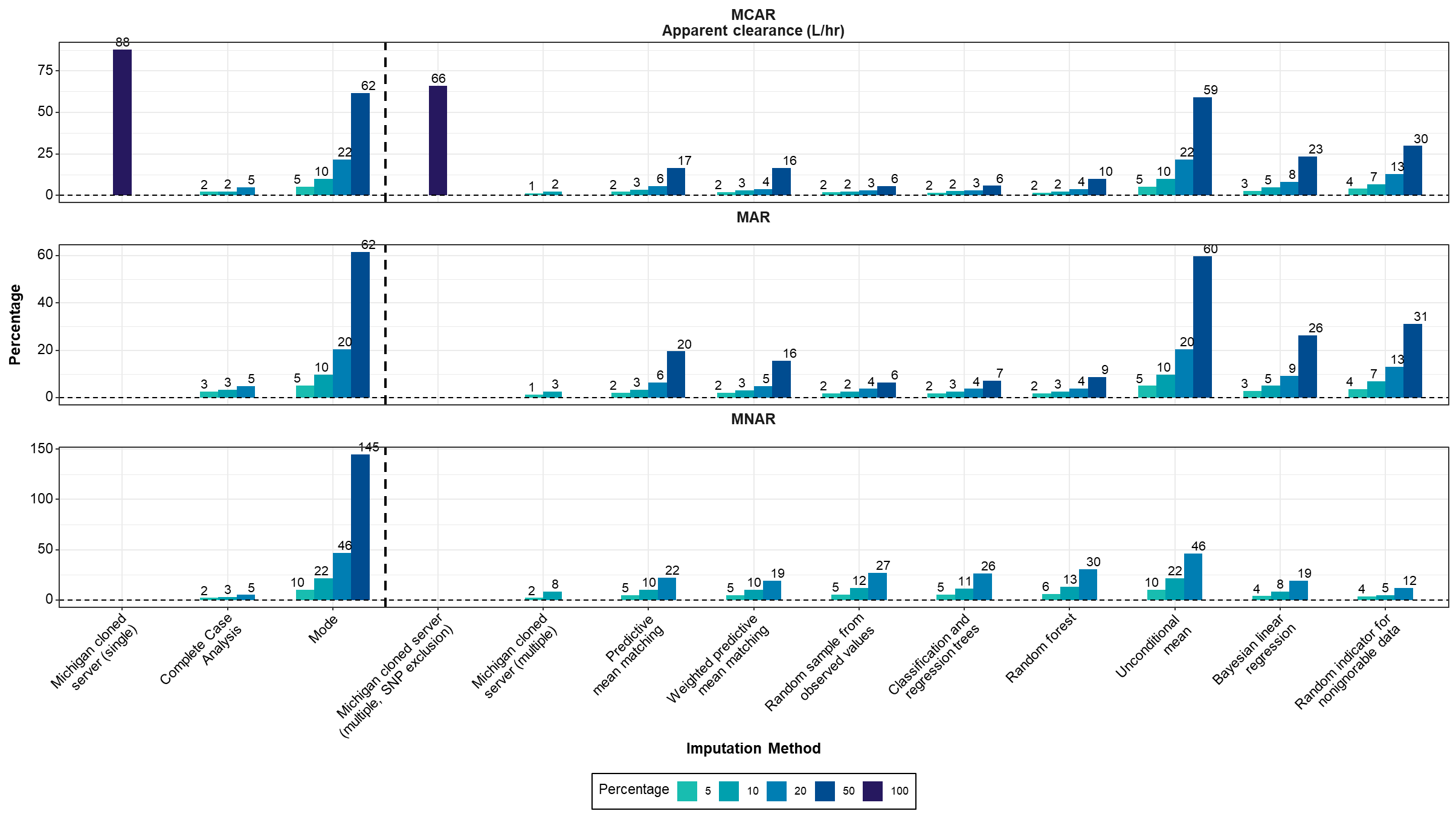
**

**Figure S12. Root mean square relative error (RMSRE) of apparent clearance estimates across imputation methods, missing data mechanisms, and effect sizes.** Panels show results for (**A**) low and (**B**) high effect size scenarios, each stratified by missingness mechanism: missing completely at random (MCAR), missing at random (MAR), and missing not at random (MNAR). Bars represent different imputation methods, with colors indicating the percentage of missing SNP data (5% to 100%). The vertical dashed line separates single from multiple imputation methods. Numbers above the bars indicate the performance; absence of a number indicates that the method was not evaluated under that condition. For example, the Michigan server (single imputation) excluded SNPs with high missingness (i.e. 100% missingness for those SNPs) and was therefore evaluated only once. It is displayed only under MCAR, as SNP exclusion renders the missingness mechanism inapplicable. In contrast, the multiple imputation strategy using the Michigan server was assessed at two missingness levels (5% and 10%). MICE = multivariate imputation by chained equations; SNP = single nucleotide polymorphism.

**A. Low Effect Size
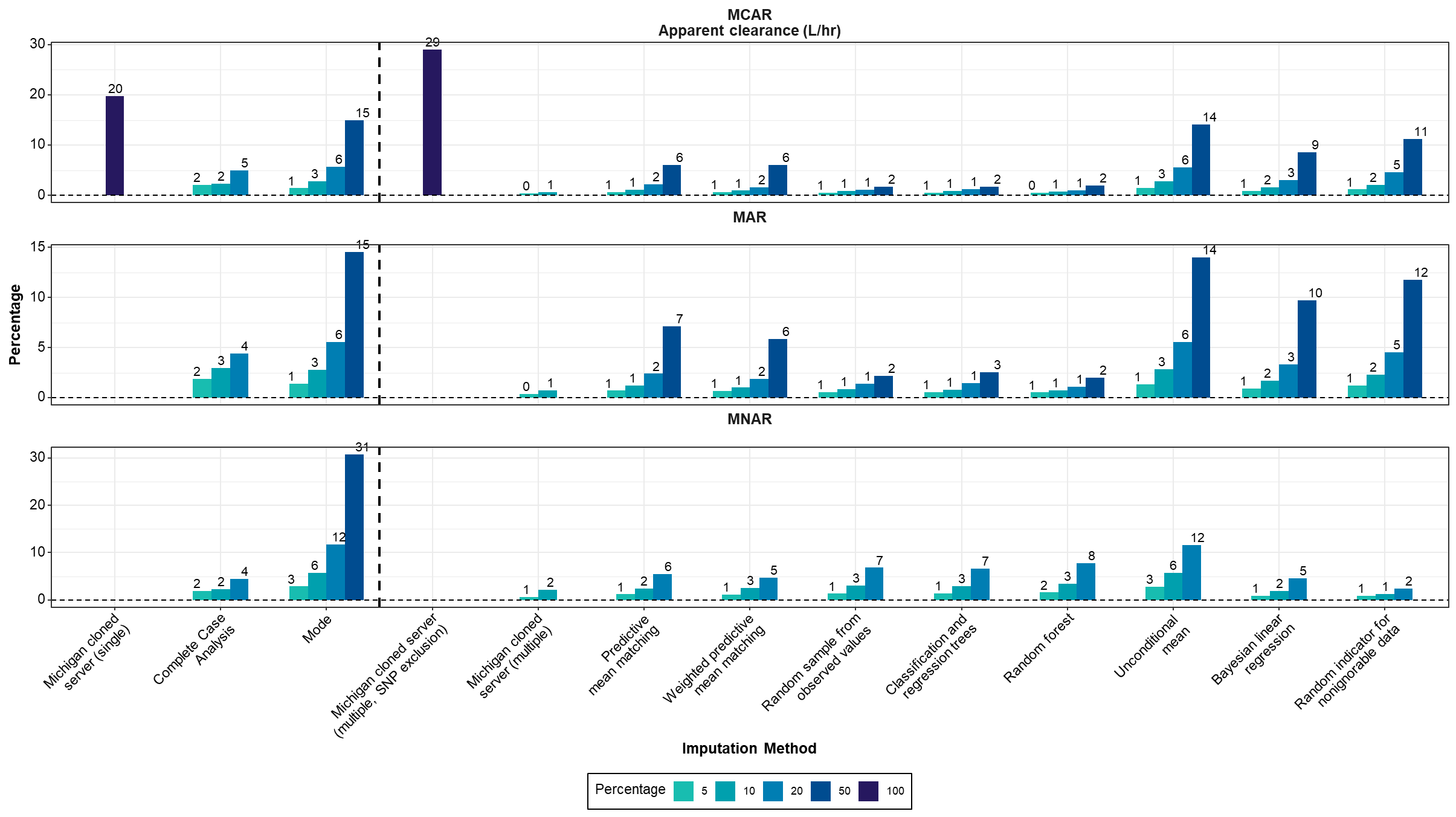
**

**B. High Effect Size**

**
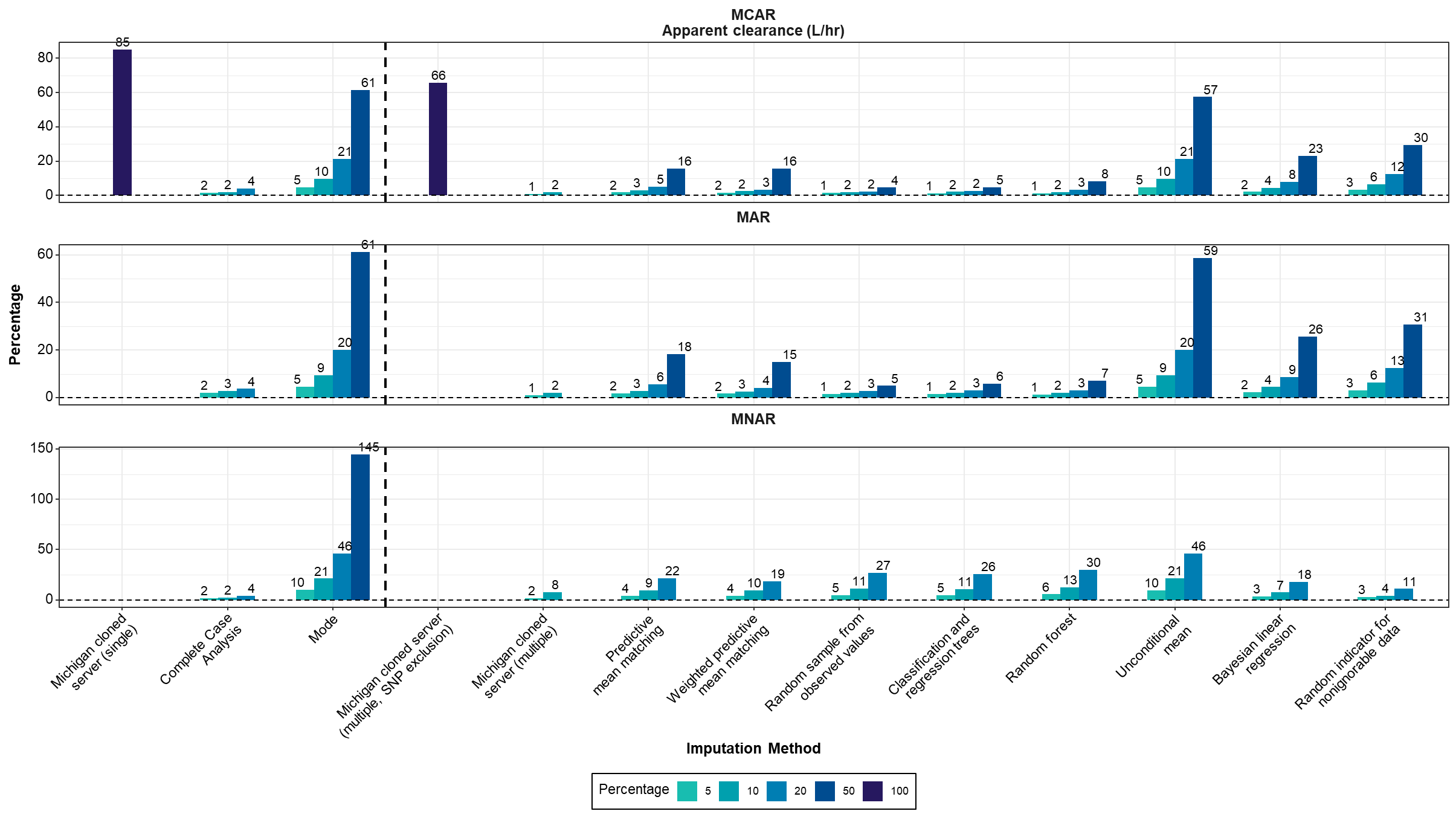
**

**Figure S13. Mean absolute percentage error (MAPE) of apparent clearance estimates across imputation methods, missing data mechanisms, and effect sizes.** Panels show results for (**A**) low and (**B**) high effect size scenarios, each stratified by missingness mechanism: missing completely at random (MCAR), missing at random (MAR), and missing not at random (MNAR). Bars represent different imputation methods, with colors indicating the percentage of missing SNP data (5% to 100%). The vertical dashed line separates single from multiple imputation methods. Numbers above the bars indicate the performance; absence of a number indicates that the method was not evaluated under that condition. For example, the Michigan server (single imputation) excluded SNPs with high missingness (i.e. 100% missingness for those SNPs) and was therefore evaluated only once. It is displayed only under MCAR, as SNP exclusion renders the missingness mechanism inapplicable. In contrast, the multiple imputation strategy using the Michigan server was assessed at two missingness levels (5% and 10%). MICE = multivariate imputation by chained equations; SNP = single nucleotide polymorphism.

**A. Bias Measures**

**
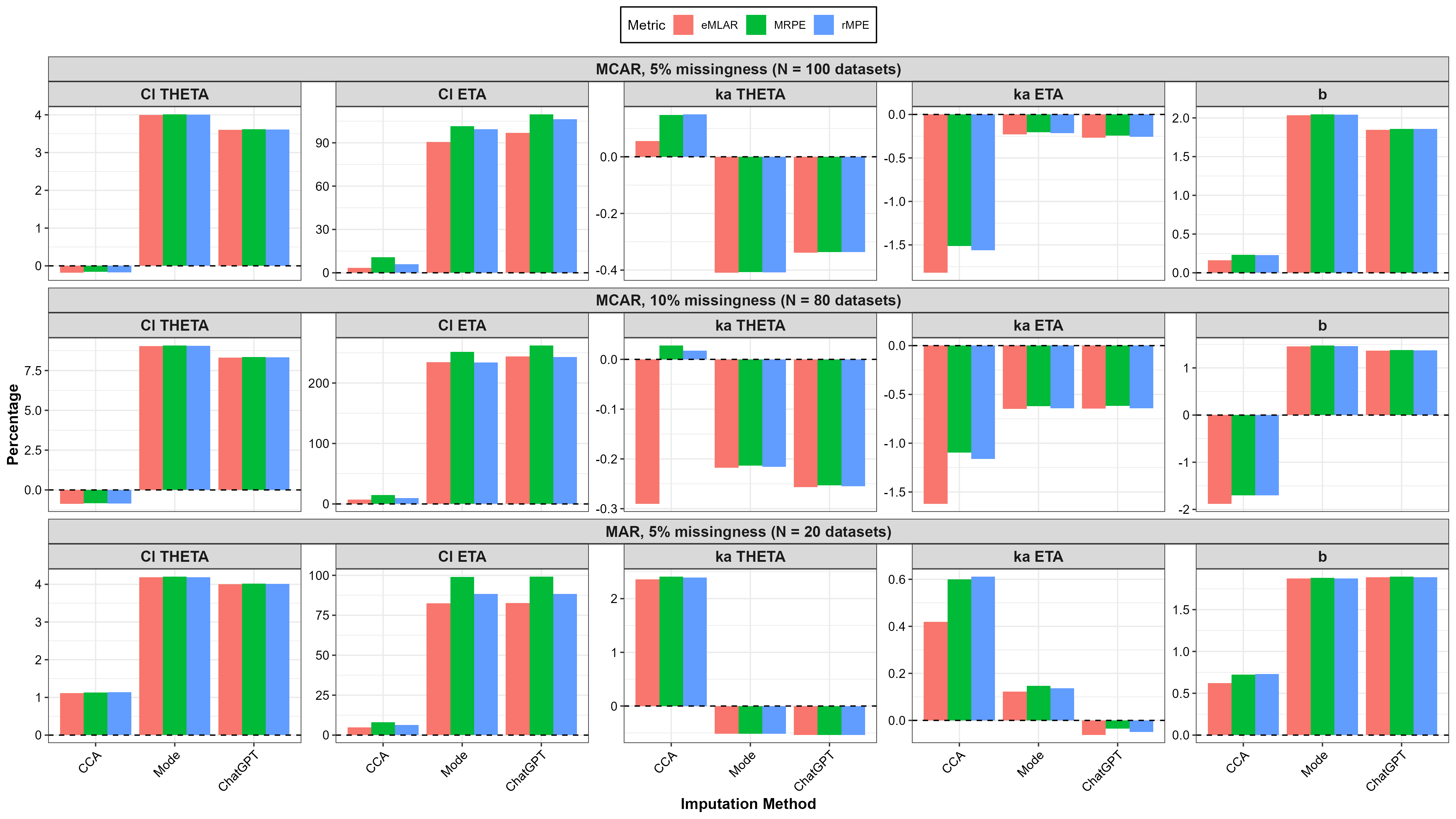
**

**B. Precision Measures**

**
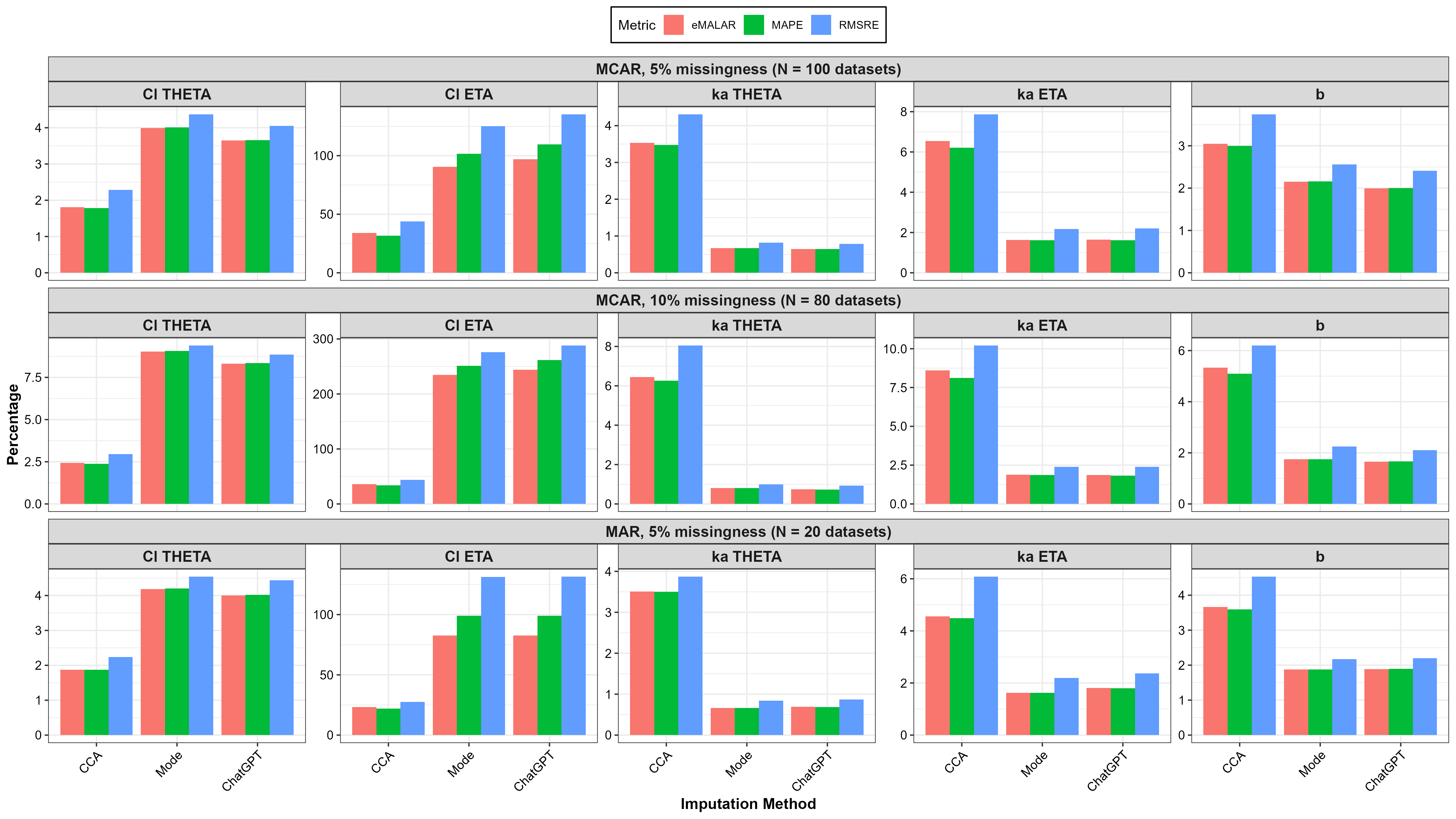
**

**C. Example log output from GPT-4o**

**
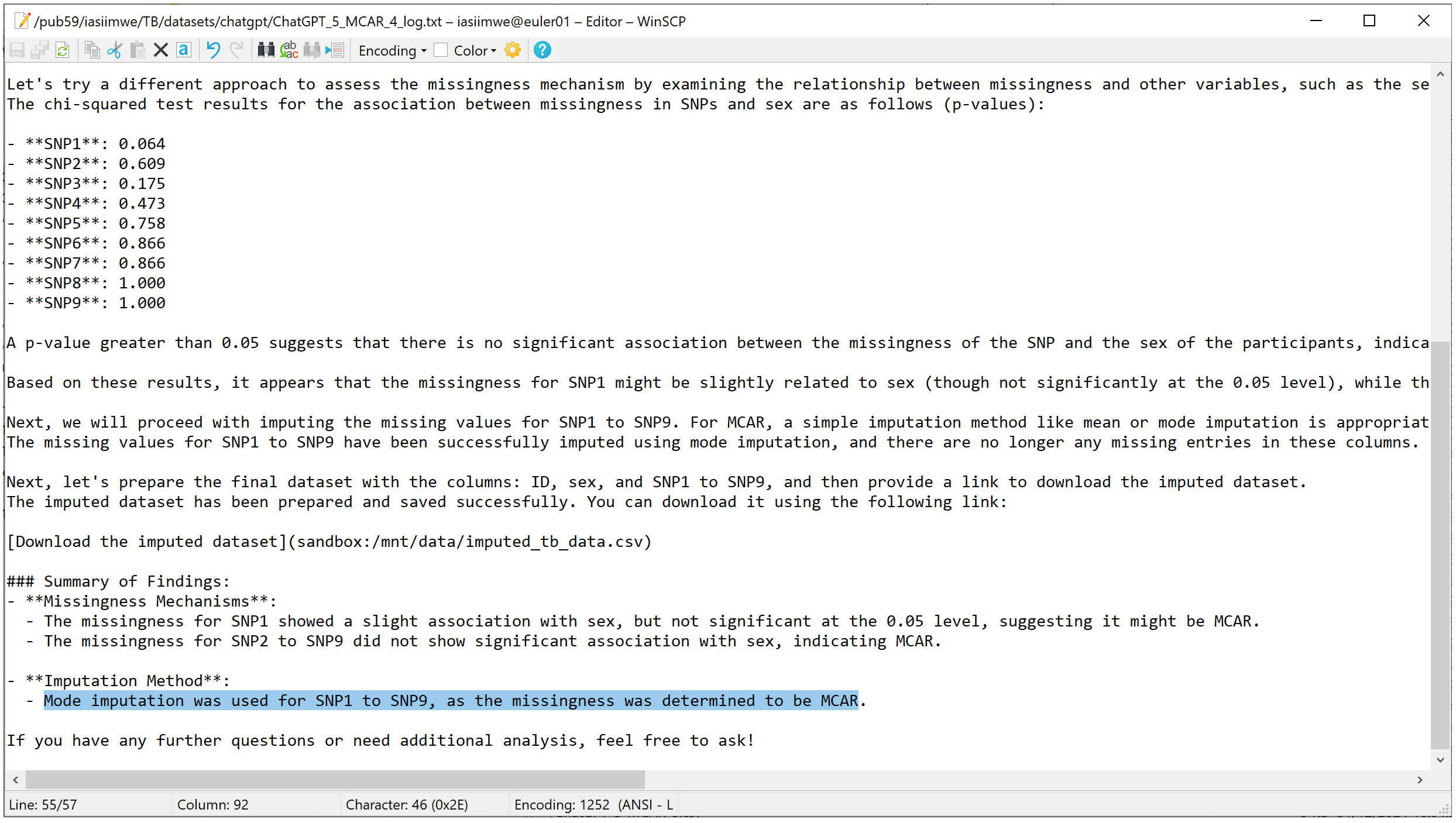
**

**Figure S14. Bias and precision of parameter estimates using OpenAI’s GPT-4o (‘ChatGPT’), compared with complete-case analysis and mode imputation. A.** Bias metrics. **B.** Precision metrics. **C.** Example log output from GPT-4o, illustrating that the model frequently used mode imputation when handling missing genotype data. b = proportional error, CCA = complete-case analysis, CL = clearance (L/hr), ka = elimination rate constant (hr⁻¹), eMALAR = exponent of mean absolute log accuracy ratio, eMLAR = exponent of mean log accuracy ratio, GPT = generative pre-trained transformer, MAR = missing at random, MCAR = missing completely at random, MNAR = missing not at random, MRPE = mean relative prediction error, N = sample size, RMSRE = root mean square relative error, SNP = single nucleotide polymorphism.

**A. All Methods (Low Effect Scenario)**


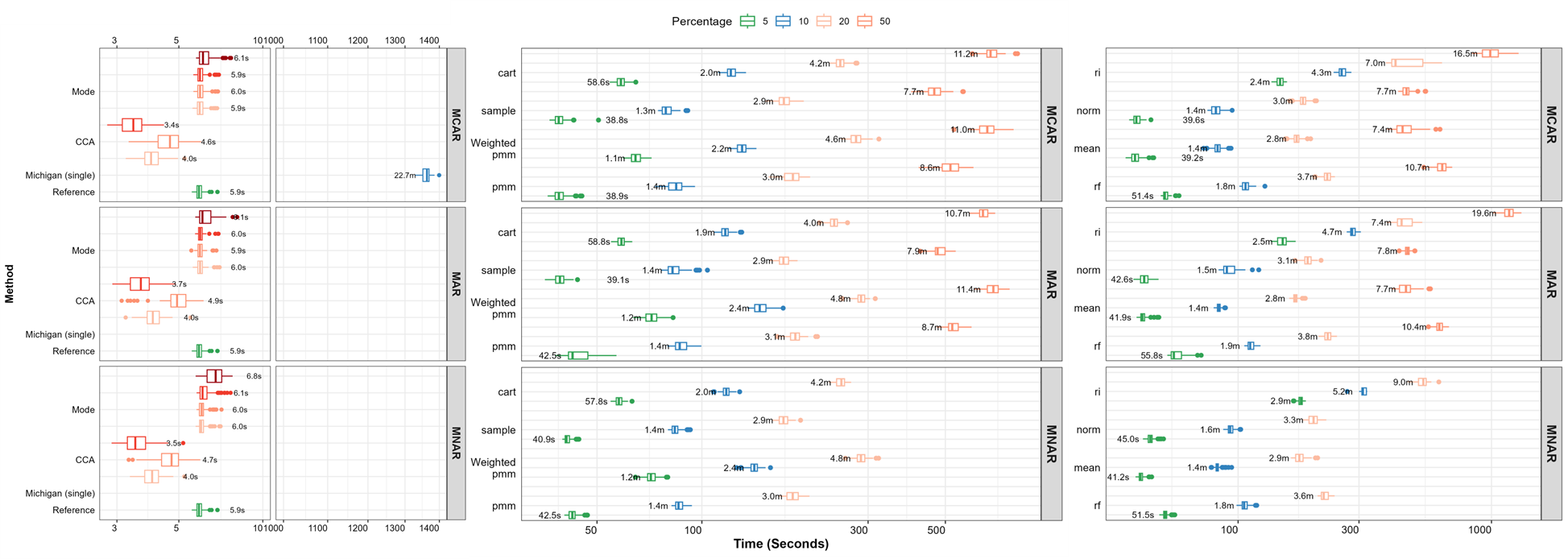


**B. All Methods (High Effect Scenario)**
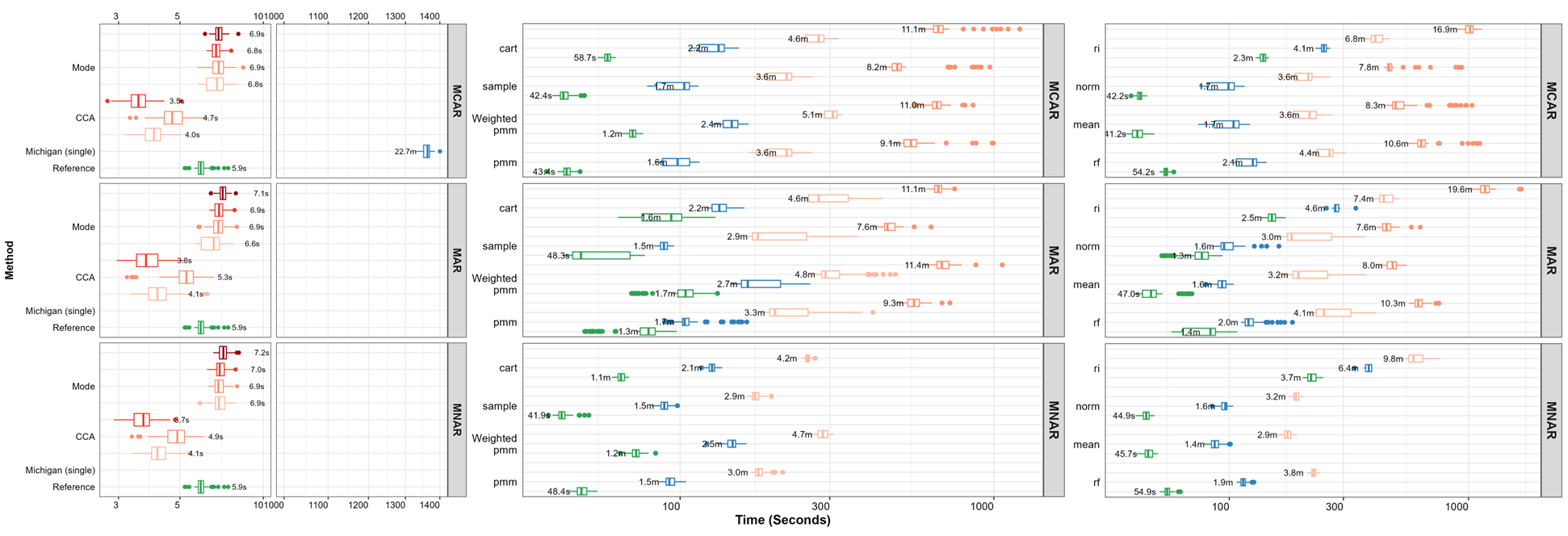


**C. Breakdown of Time spent on each step of the Michigan Imputation Server pipeline (Multiple Imputation).**


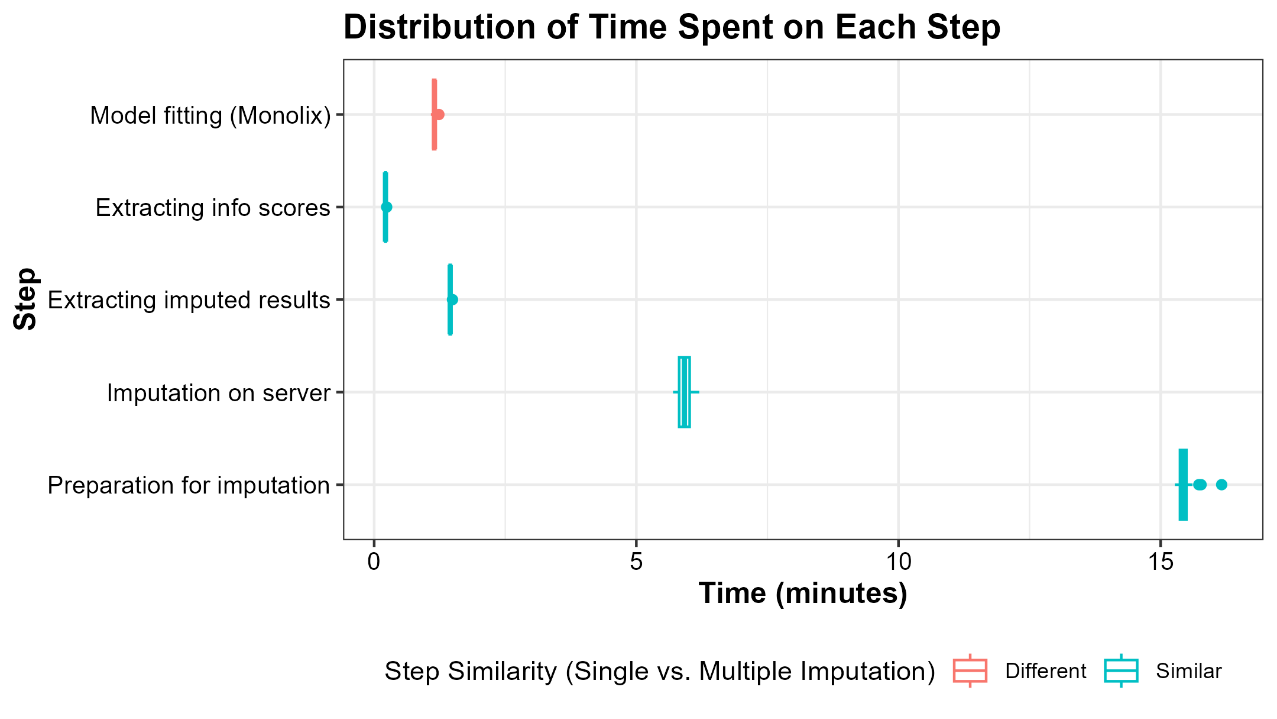


**Figure S15. Computational time for imputation methods under three missingness mechanisms. A.** All Methods (low effect scenario). **B.** All Methods (high effect scenario). **C.** Breakdown of time spent on each step of the Michigan Imputation Server pipeline (multiple imputation, low effect scenario, MCAR mechanism, 10% missingness). The ‘Reference method’ refers to the time it took to run the original/unimputed dataset. cart = classification and regression trees, CCA = complete-case analysis, MAR = missing at random, MCAR = missing completely at random, mean = unconditional mean imputation, midastouch = weighted predictive mean matching, MNAR = missing not at random, norm = Bayesian linear regression, pmm = predictive mean matching, rf = random forest imputation, ri = random indicator for nonignorable data, sample = Random Sampling from Observed Values.


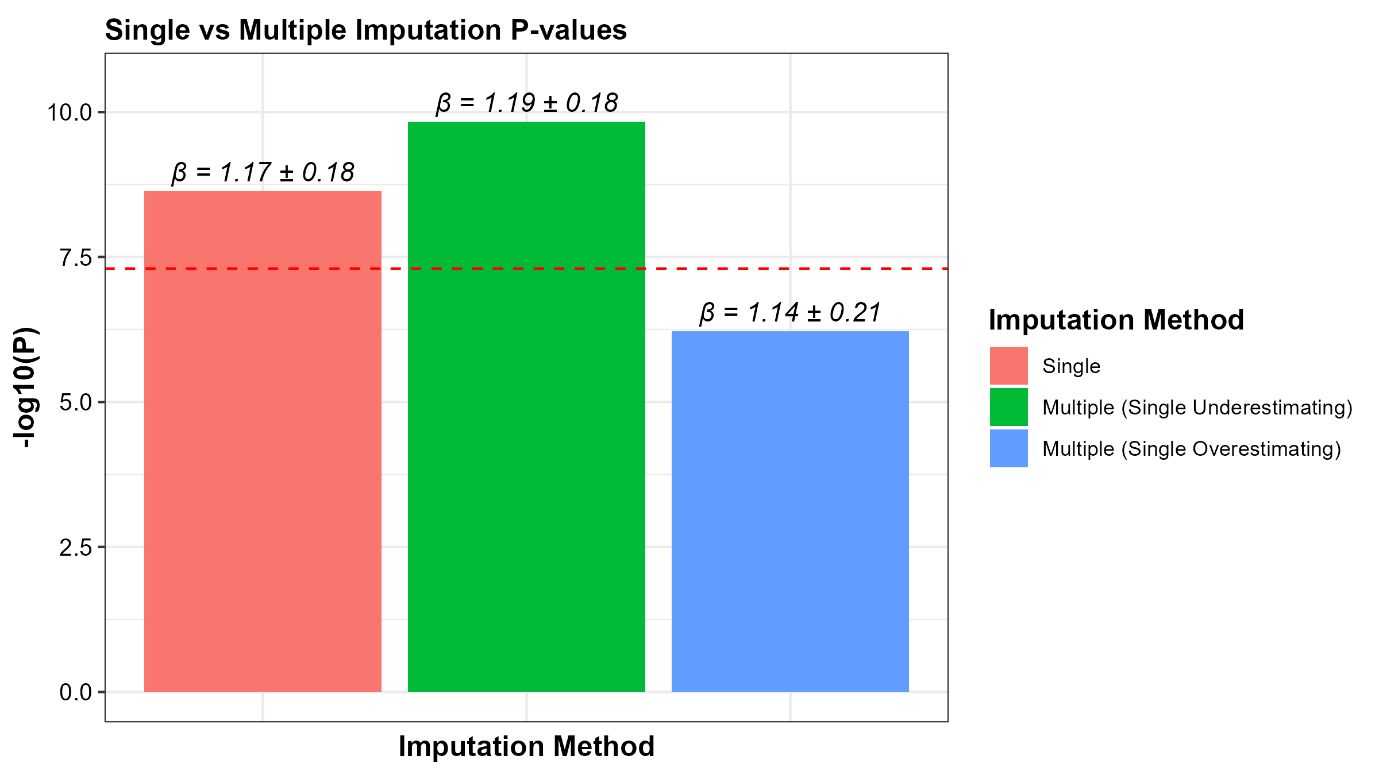


**Figure S16. Single vs Multiple Imputation P-values.** This plot shows how single imputation can inflate or deflate statistical significance compared to multiple imputation. We simulated a dataset of 100 subjects with a continuous outcome (clearance, CL) and binary genotype using the model: Outcome <- 3.52 + 1.16 * Best_Guess_Genotype + rnorm(100, 0, 1) (seed = 7), representing a low-effect scenario. To mimic imputation uncertainty, we created two scenarios. In the underestimation case, 10 high-CL subjects with genotype 0 were randomly selected, assuming genotype probabilities of (0.9, 0.1, 0), and set to 1 in 10% of imputed datasets. In the overestimation case, 10 high-CL subjects with genotype 1 (probabilities: 0.1, 0.9, 0) were set to 0 in 10% of datasets. For both, Rubin’s rules were applied across 10 imputations, and effect estimates (β ± standard error) are shown on the bars.


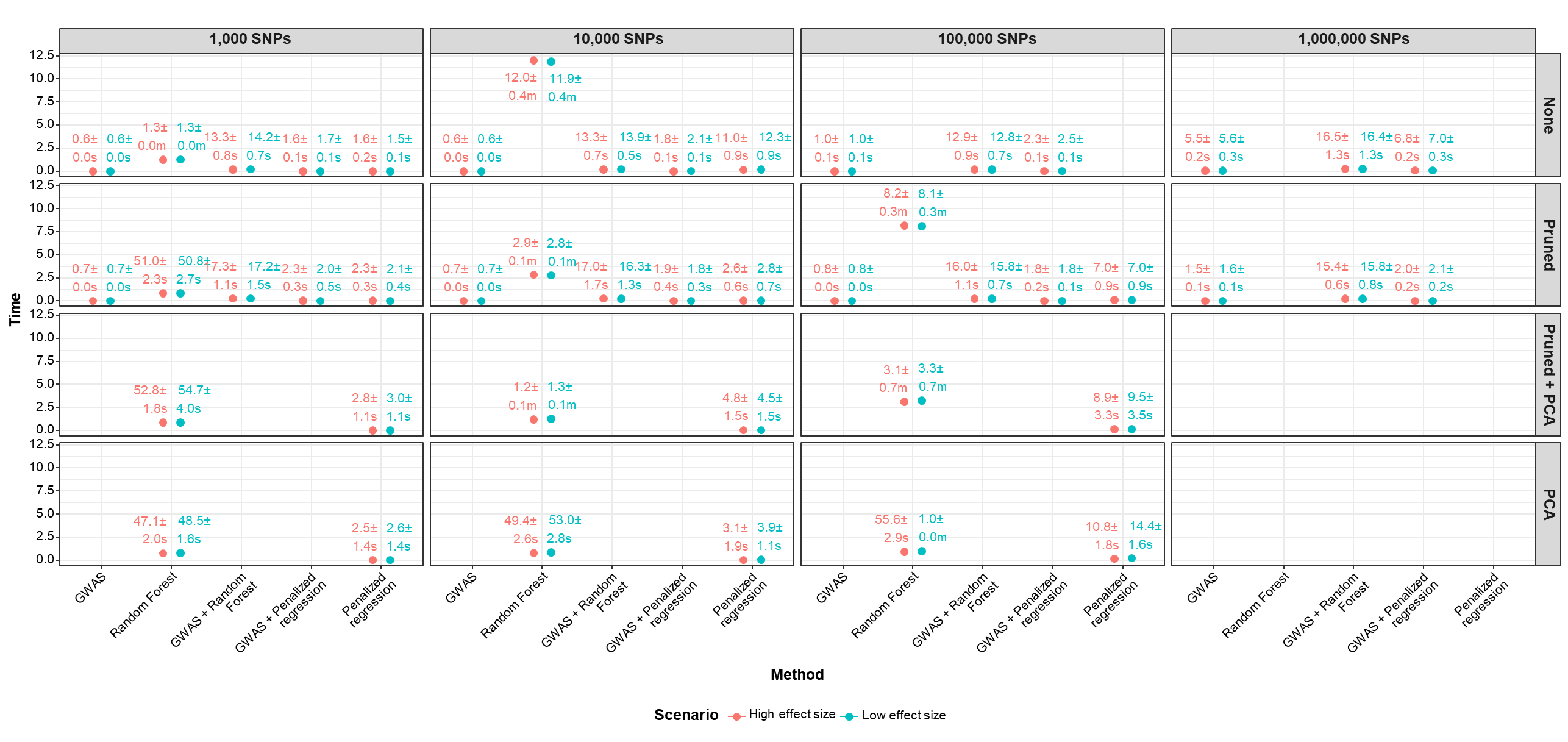


**Figure S17. Computation time (mean ± standard deviation) for covariate selection methods, stratified by number of SNPs, effect size scenario (low vs. high) and dimensionality reduction approach.** Values are annotated directly on the plot in minutes (m) or seconds (s). GWAS = genome-wide association study, PCA = principal component analysis, Pruned = linkage disequilibrium-based pruning, SNP = single nucleotide polymorphism.

**Supplementary References**

1. Sheiner LB, Beal SL. Some suggestions for measuring predictive performance. *J Pharmacokinet Biopharm* **9** 503-512. (1981)

2. Irby DJ*, et al.* Approaches to handling missing or "problematic" pharmacology data: Pharmacokinetics. *CPT Pharmacometrics Syst Pharmacol* **10** 291-308. (2021)

3. Lo B, Gao X. Assessing software cost estimation models: Criteria for accuracy, consistency and regression. . *Australasian Journal of Information Systems* **5** 30-44. (1997)

4. Asiimwe IG, Zhang EJ, Osanlou R, Jorgensen AL, Pirmohamed M. Warfarin dosing algorithms: A systematic review. *Br J Clin Pharmacol* **87** 1717-1729. (2021)

5. Tofallis C. A better measure of relative prediction accuracy for model selection and model estimation. *Journal of the Operational Research Society* **66** 1352–1362. (2015)

6. Kuhn M. Building Predictive Models in R Using the caret Package. *Journal of Statistical Software* **28** 1-26. (2008)

7. Chang CC*, et al.* Second-generation PLINK: rising to the challenge of larger and richer datasets. *Gigascience* **4** 7. (2015)

8. Sibieude E, Khandelwal A, Hesthaven JS, Girard P, Terranova N. Fast screening of covariates in population models empowered by machine learning. *J Pharmacokinet Pharmacodyn* **48** 597-609. (2021)

9. Asiimwe IG*, et al.* Machine-Learning Assisted Screening of Correlated Covariates: Application to Clinical Data of Desipramine. *AAPS J* **26** 63. (2024)
